# Nuclear and cytoplasmic RNA exosomes and PELOTA1 prevent miRNA-induced secondary siRNA production in Arabidopsis

**DOI:** 10.1093/nar/gkab1289

**Published:** 2022-01-17

**Authors:** Maria L Vigh, Simon Bressendorff, Axel Thieffry, Laura Arribas-Hernández, Peter Brodersen

**Affiliations:** University of Copenhagen, Copenhagen Plant Science Center, Ole Maaløes Vej 5, 2200 Copenhagen N, Denmark; University of Copenhagen, Copenhagen Plant Science Center, Ole Maaløes Vej 5, 2200 Copenhagen N, Denmark; University of Copenhagen, Copenhagen Plant Science Center, Ole Maaløes Vej 5, 2200 Copenhagen N, Denmark; University of Copenhagen, Copenhagen Plant Science Center, Ole Maaløes Vej 5, 2200 Copenhagen N, Denmark; University of Copenhagen, Copenhagen Plant Science Center, Ole Maaløes Vej 5, 2200 Copenhagen N, Denmark

## Abstract

Amplification of short interfering RNA (siRNAs) via RNA-dependent RNA polymerases (RdRPs) is of fundamental importance in RNA silencing. Plant microRNA (miRNA) action generally does not involve engagement of RdRPs, in part thanks to a poorly understood activity of the cytoplasmic exosome adaptor SKI2. Here, we show that inactivation of the exosome subunit RRP45B and SKI2 results in similar patterns of miRNA-induced siRNA production. Furthermore, loss of the nuclear exosome adaptor HEN2 leads to secondary siRNA production from miRNA targets largely distinct from those producing siRNAs in *ski2*. Importantly, mutation of the Release Factor paralogue PELOTA1 required for subunit dissociation of stalled ribosomes causes siRNA production from miRNA targets overlapping with, but distinct from, those affected in *ski2* and *rrp45b* mutants. We also show that in exosome mutants, miRNA targets can be sorted into producers and non-producers of illicit secondary siRNAs based on trigger miRNA levels and miRNA:target affinity rather than on presence of 5′-cleavage fragments. We propose that stalled RNA-Induced Silencing Complex (RISC) and ribosomes, but not mRNA cleavage fragments released from RISC, trigger siRNA production, and that the exosome limits siRNA amplification by reducing RISC dwell time on miRNA target mRNAs while PELOTA1 does so by reducing ribosome stalling.

## INTRODUCTION

Small RNA-guided repression of mature mRNA involves a pathway that in outline is simple and linear. Dicer ribonucleases process RNA with double-stranded features into 20–24-nt duplexes with 2-nt 3′-overhangs, and one of the two strands stably associates with an Argonaute (AGO) protein to form a mature, minimal RNA-induced Silencing Complex (RISC). RISC then uses the base pairing specificity of the small RNA to find complementary targets, and it may use the endonuclease activity of AGO to cleave target RNA, the process referred to as slicing (1,2). The employment of this simple module gives rise to repression that can be relieved by degradation or arrest of biogenesis of the miRNA. In plants, nematodes and many fungi, complexity may be added via a positive feedback module, as RNA-dependent RNA Polymerases (RdRPs) may be recruited to RNAs targeted by RISC loaded with a primary small RNA species (3,4). In nematodes, direct RdRP products guide target RNA silencing (5), whereas in plants, RdRP activity gives rise to synthesis of target-templated double-stranded RNA (dsRNA) that serves as substrate for DICER-LIKE proteins to generate more small interfering RNAs (siRNAs) complementary to the target (6–8). These amplified siRNAs are referred to as secondary siRNAs, and reiterative rounds of amplification may then generate tertiary siRNAs, quaternary siRNAs, etc. In plants, single-round amplification produces a diagnostic phased pattern of siRNA accumulation resulting from consecutive dicing of dsRNA with a well-defined end produced by RISC-catalyzed slicing (9–11). Regardless of the exact mechanism of secondary small RNA biogenesis, the decision to employ small RNA amplification represents a major checkpoint of genetic control because it determines the resulting type of regulation. In plants, three fundamentally different outcomes are possible depending on whether and how small RNA amplification is employed. First, in the total absence of amplification, silencing is reversible and dependent on the continued production of the primary small RNA species. In addition, in the case of miRNAs, the regulation is typically cell-autonomous or restricted to spreading over a few cells by cell-to-cell movement (12–15), although important examples of miRNA action over long distances via phloem transport have also been reported (16,17). Second, if a single round of secondary siRNAs is produced, gene regulation remains reversible and dependent on the primary trigger (18), but the regulatory siRNAs move efficiently from cell to cell (15). This phenomenon has profound biological ramifications as it may result in formation of concentration gradients and consequent action of siRNAs as morphogens during development (19,20), and in vesicle-mediated siRNA-transfer into fungal and oomycete pathogens as part of immune responses (21,22). The typical example of such small RNAs resulting from single amplification rounds is *trans-*acting siRNAs (tasiRNAs), whose accumulation pattern in phase with a miRNA-directed cleavage site is proof of single-round amplification (3,7,8). Third, if reiterative amplification is produced, silencing is effectively irreversible as it becomes independent of the primary trigger and maintains the target silenced. Endogenous transcripts are typically subjected to the first two reversible outcomes of silencing, while the third option is used to silence foreign RNA, for example transgenic, transposon or viral RNA (23–28). Thus, the employment of the RdRP amplification module represents a central decision in gene regulation and is tightly linked to the fundamental problem of distinguishing self- from non-self RNA.

Although the RdRP RDR6 was the first factor required for small RNA-guided transgene silencing to be identified in plants (23,24), only few fundamental questions regarding its engagement in production of dsRNA substrates for Dicers have been answered. Careful examination of its substrate preferences *in vitro* showed that RDR6 activity is nearly completely inhibited by a 3′ stretch of >8 adenosines (29), thus providing a simple explanation for how functional, polyadenylated mRNAs escape use as an RDR6 substrate. On the other hand, while it is now clear that 22-nt, but not 21-nt, miRNAs tend to trigger RDR6-dependent, secondary siRNA production (30–32), there is no clear explanation for why the two different size classes of miRNAs exhibit this fundamentally different behavior. The most fruitful way to put this question may be to ask which mechanisms allow cells to avoid secondary siRNA production in target interactions involving 21-nt miRNAs, and which properties of 22-nt miRNAs cause those protective mechanisms to be overridden.

Studies of small RNA-production in mutants with lesions in key factors of mRNA decay, such as core components or activators of decapping, the 5′-3′ exoribonuclease XRN4 (33–38), or the major 3′-5′-exoribonuclease complex, the RNA exosome (39–41), have shown that a wide range of mRNAs become sources of RDR6-dependent siRNAs in these backgrounds. Such results are generally interpreted as support for the model that aberrant RNA may initiate RDR6-dependent siRNA production. Because the sets of mRNAs giving rise to siRNA production in these mutants are generally not significantly enriched in miRNA targets, or the ectopic siRNAs were not analyzed with an eye toward avoidance of miRNA-triggered siRNA production, these studies do not provide useful answers to the problem of how endogenous miRNA targets escape RDR6-dependent siRNA formation.

Our previous work demonstrated that one mechanism of avoiding illicit RDR6-dependent secondary siRNAs induced by 21-nt miRNAs in *Arabidopsis* involves the DExH-type RNA helicase SUPERKILLER2 (SKI2) because a limited number of mRNAs strongly enriched in known miRNA targets accumulates secondary siRNAs mapping close to miRNA binding sites in *ski2* mutants (42). SKI2 is part of the heterotetrameric, cytoplasmic SKI2-3-8 complex that acts as an adaptor for the RNA exosome (43–45). Consistent with this biochemical function, mutation of each of *SKI2*, *SKI3* and *SKI8* results in accumulation of stable RISC 5′-cleavage fragments from several miRNA targets (42).

Cleaved mRNAs without stop codons, resulting in stalled ribosomes with empty A-sites constitute an important class of substrates of the SKI2-SKI3-SKI8-exosome pathway (46,47). In animals, this so-called non-stop decay (NSD) pathway depends on recognition of stalled ribosomes at or close to mRNA 3′-ends by the Release Factor (RF)-like proteins Pelota (in yeast referred to as Dom34) and Hbs1 (48,49) that cause ribosome subunit dissociation and release of intact peptidyl-tRNA to achieve ribosome recycling (50). 5′-cleavage fragments generated through miRNA-guided cleavage are generally NSD substrates because most miRNA binding sites occur in open reading frames in plants (51–53). Indeed, studies of the *Arabidopsis* orthologues of Pelota and Hbs1 showed that inactivation of these NSD factors also results in accumulation of stable RISC 5′-cleavage fragments, similar to mutation of *SKI2*, with plant PELOTA being indispensable and HBS1 playing an accessory role (54). The relative importance of PELOTA and HBS1 is similar to what was previously observed for ribosome subunit dissociation from stalled elongation complexes in other eukaryotic systems (55). The *Arabidopsis* studies also revealed that plants encode two PELOTA homologues, PEL1 and PEL2, and that PEL1 is the functional homologue of metazoan Pelota, while PEL2 appears to act as a dominant negative NSD factor, perhaps through its intact HBS1-binding activity (56).

Given the lack of a 3′ poly(A)-tail of 5′-cleavage fragments and the inhibitory activity of oligo(A) tracts on RDR6 activity, combined with genetic evidence for the implication of aberrant RNA in siRNA production, it is tempting to speculate that mutation of *SKI2* indirectly allows secondary siRNA formation via action of RDR6 on stable 5′-cleavage fragments. Indeed, abundant siRNAs mapping to miRNA targets were noted among the many ectopic siRNA species detected in *xrn4*/*ski2* double mutants, and defective elimination of aberrant RNA produced by RISC-mediated cleavage in the case of miRNA targets was proposed as the mechanism underlying RDR6-mediated siRNA production (57). This seemingly straight-forward interpretation is in conflict with several observations, however. First, for some miRNA targets, stable 5′-cleavage fragments, but no secondary siRNAs, can be detected in *ski2* mutants (42). Second, for other miRNA targets, secondary siRNAs accumulate exclusively 3′ to the cleavage site despite clear accumulation of 5′-cleavage fragments, precluding action of RDR6 on stable 5′-cleavage fragments as the general mechanism of illicit secondary siRNA formation in *ski2* mutants (42). Thus, although SKI2 is part of one of the molecular mechanisms that guards against siRNA amplification induced by 21-nt miRNAs, it is unclear how it does so. A key prerequisite for a better understanding of this important problem is to clarify whether the two effects of SKI2 rely on the same biochemical activity—acceleration of exosome-mediated degradation of 5′-cleavage fragments—or whether SKI2 uses a separate activity to ensure avoidance of secondary siRNA production. We reasoned that if the RNA decay activity of the SKI2/exosome pathway is implicated in avoiding miRNA-triggered secondary siRNAs, mutants in all components of this pathway should show defects in limiting secondary siRNA production similar to *ski2* mutants. On the other hand, this defect should be specific to *ski2* mutants if a function of the protein distinct from stimulation of exosome-coupled RNA decay limits secondary siRNA production.

We show here that mutants in *SKI3* and in the exosome subunit encoded by the *RRP45B* paralogue have defects in limitation of secondary siRNA production very similar to *ski2* mutants, and that a hypomorphic mutant in the gene encoding the core exosome subunit RRP4 has more widespread defects in limiting production of illicit siRNAs. Mutants in *RRP45B* also have defects in degradation of RISC 5′-cleavage fragments, thus indicating that SKI2-mediated exosomal decay of 5′-cleavage fragments is necessary for avoidance of miRNA-triggered secondary siRNAs. Furthermore, inactivation of the nuclear exosome cofactor *HEN2* leads to secondary siRNA generation from a subset of miRNA targets largely distinct from those affected by SKI2, SKI3, and RRP45B, perhaps suggesting that some miRNA targets may undergo RISC-mediated cleavage in the nucleus. We also show that the expression level of 21-nt miRNAs and their affinity for target mRNAs, not the accumulation of their RISC-mediated cleavage fragments, correlate with their ability to trigger secondary siRNAs in *rrp4* mutants. We discuss these results in the light of our observation in the present work that mutation of *PEL1* leads to miRNA-induced siRNA amplification similar, but not identical, to that observed in *ski2*, *ski3* and *rrp45b*, and of recent evidence for the importance of ribosome stalling for siRNA production (58).

## MATERIALS AND METHODS

### Plant material and growth conditions

All mutants used in this study are of the ecotype Columbia (Col-0). T-DNA insertion mutants in *SKI2* (AT3G49690: *ski2-2* (SALK_129982), *ski2-5* (SALK_118529)), *SKI3* (AT1G76630: *ski3-5* (GK_140B07))*, SKI8* (AT4G29830: *ski8-1* (SALK_083364)), *HEN2* (AT2G06990: *hen2-4* (SALK_091606), *hen2-5* (GK_313G10_015818)), *PEL1* (AT4G27650: *pel1-1* (SAIL_881_B10)), RRP45A (AT3G12990: *rrp45a* (GK_655_D02)), *RRP45B* (AT3G60500: *cer7-3* (SAIL_747_B08) and *cer7-3*/*rdr6-12*)) and *SOP1* (AT1G21580: *sop1-*5 (SALK_019457) have been described previously (42,54,59–63), as has the *rrp4-2/sop2* point mutant allele (61). *pel1-2* (GK_537F08) was identified from the GABI-KAT collection of T-DNA insertion mutants (64). All T-DNA mutants except *cer7-3*, *cer7-3*/*rdr6-12*, *hen2-4*, *hen2-5* and *sop1-5* were obtained from the Nottingham Arabidopsis Stock Centre*. cer7-3* and *cer7-3*/*rdr6-12* mutant seeds were a gift from Ljerka Kunst, *hen2-4* mutant seeds were a gift from Dominique Gagliardi, while *hen2-5*, *sop1-5* and *rrp4-2* mutant seeds were a gift from Kian Hématy.

All experiments were carried out with inflorescence tissue. To obtain this tissue, seedlings were germinated on plates containing 1× Murashige & Skoog medium (#M0222.0050, Saveen o Werner ApS, Denmark), supplemented with 1% (w/v) sucrose and 0.8% (w/v) agar, transferred to soil (Plugg/Såjord [seedcompost]; SW Horto, Bramming, Denmark) 10 days after germination (DAG) and grown in a Percival growth chamber for an additional 4 weeks under the following conditions: 16 h light (Master TL-D 36W/840 and 18W/840 bulbs (Philips); 130 mmol m^−2^ s^−1^, 21°C, 60% relative humidity)/ 8 h darkness (16°C, 60% relative humidity). Inflorescences were collected and snap-frozen in liquid nitrogen.

### Mutant genotyping

Each T-DNA insertion mutant was genotyped with two PCR reactions: a PCR reaction with a set of primers flanking the insertion site for wild type allele detection and a PCR reaction with an outward left border primer and one of the two flanking primers for T-DNA allele detection. Genotyping of *rrp4-2* was carried out with a single PCR reaction with primers spanning the point mutation followed by restriction analysis (Eco47I, (#ER0311, Thermo Scientific™)). Genotyping of *rdr6-12* was carried out as described in (11).

For construction of the *hen2-4/ski2-5* double mutant, F2 populations generated by self-pollination of F1 plants obtained from *hen2-4* crossed to *ski2-5* were genotyped as described above to find double homozygous mutants. In an attempt to construct a *ski2/pel* double mutant, *pel1-1* and *pel1-2* were crossed to *ski2-5* and *pel1-2* was in addition crossed to *ski2-2*. F2 populations generated by self-pollination of F1 plants were genotyped, but no double mutant was identified from any of the three crosses (96 seedlings from each *ski2/pel1* cross combination were genotyped). Siliques from *ski2-5 pel1-2/+, ski2-5 pel1-1/+* and *pel1-2 ski2-2/+* plants, identified during genotyping of the F2 population, were cut open and presence of aborted seeds was verified and documented by photography and by assessment of the frequency of aborted seeds.

All primers used in this study were purchased from TAG Copenhagen A/S and their sequences are listed in Supplementary Table S1.

### RNA extraction

Total RNA from inflorescences was extracted with TRI Reagent (#T9424, Sigma-Aldrich Denmark A/S) according to the manufacturer’s instructions. The RNA was dissolved in either 50% formamide for northern blots or in sterilized water for small RNA libraries and RT-PCR analysis.

### Northern blotting of mRNA cleavage fragments

20 μg of purified total RNA was loaded onto a 1% denaturing agarose gel and separated for 3 h at 120 V. The RNA was blotted to an Amersham Hybond-NX membrane (#RPN303, GE Healthcare Life Sciences) followed by UV-crosslinking (254 nm). Two gels with the same batch of extracted RNA were prepared in parallel. After pre-hybridization in PerfectHyb™ Plus Hybridization buffer (#H7033, Sigma-Aldrich Denmark A/S) for 1 h at 65°C, the blots were hybridized O/N at 65°C to radioactively labeled 3′-CF probes produced with Prime-a-gene labeling kit (#U1100, Promega). The membranes were washed 3 times in 2× SSC (0.3 M NaCl, 30 mM sodium citrate), 0.1% SDS at 65°C and developed by phosphorimaging. The blots were subsequently stripped with boiling 0.1% SDS and rehybridized with each their radioactively labeled 5′-CF probes. Primers used for amplification of probe templates of AGO1, ARF10, CSD2, PHO2 and SPL2 cleavage fragments were purchased from TAG Copenhagen A/S and their sequences are listed in Supplementary Table S1. The RNA for Northern blotting was extracted from a pool of inflorescences from three plants.

### Quantitative RT-PCR (qPCR) analysis

2 μg of purified total RNA was DNAse-treated with 1 U of DNAse I (#EN0521, Thermo Scientific™). cDNA was obtained by incubation of the DNAse-treated RNA with 5 μM of Random Hexamer Primers (#SO142, Thermo Scientific™) and 200 U of RevertAid Reverse Transcriptase (#EP0441, Thermo Scientific™). After verification of cDNA production by PCR amplification of an ACTIN2 fragment, qPCR reactions were prepared with Maxima SYBR Green/ROX solution (#K0222, Thermo Scientific™). All qPCR reactions were run with 40 cycles of 2-step PCR with both annealing and elongation temperatures of 60°C. The same RNA as for northern blotting was used in the qPCR experiment. Therefore, the qPCR results are based on three technical replicates of RNA from inflorescences pooled from three plants. Except for RSG2, primers used in the qPCR reactions are spanning the miRNA cleavage site to ensure full-length mRNA amplification. For RSG2, the amplicon is located 3′ to the cleavage site, so that the RSG2 qPCR assays takes both full-length mRNA and 3′-cleavage fragments into account. Analysis of the relative gene expression was based on ΔΔ*C*_t_ calculations and statistical testing was performed with an ANOVA followed by a Tukey test for each of the three targets. All qPCR primers were purchased from TAG Copenhagen A/S and their sequences are listed in Supplementary Table S1.

### Organization of small RNA sequencing experiments

This study includes analysis of raw small RNA sequencing data from three independent sets of experiments, referred to as experiments A, B and C. sRNA sequencing data from experiment A (Col-0 WT, *ski2-5, ski3-5, rrp45b*) is used in Figure 2. sRNA sequencing data from experiment B (Col-0 WT, *ski2-5, pel1-1, pel1-2, hen2-5, rrp4, rrp45a*) is used in Figures 3–7. sRNA sequencing data from experiment C (Col-0 WT, *ski2-5, hen2-5, sop1-5*) is used in Supplementary Figure S6. Experiment A includes two biological replicates of each genotype, while experiment B and C were performed using three biological replicates for each genotype, except for the genotype *pel1-1*, which is represented in experiment B with one biological sample due to loss of tissue during sampling. Each biological replicate represents inflorescences collected from four individual plants of the same genotype grown in the same pot.

### Construction of libraries for small RNA sequencing

1 μg of total inflorescence RNA was used as input in each library. Libraries were generated using the NEBNext Multiplex Small RNA Library Prep Set (#E7300S, New England Biolabs) according to the manufacturer’s instructions. The indexed cDNA libraries were size selected on a 6% polyacrylamide gel as described in the NEBNext protocol. All size-selected libraries were analyzed using an Agilent Bioanalyzer, quantified with Qubit measurements (#Q32854, Invitrogen) and single-end sequenced on an Illumina Nextseq 500 with SE75_HI chemistry (#FC-404-2005, Illumina). 1% of spike-in PhiX Control v3 (#15017666, Illumina) was also loaded on the flow cells in each of the three runs.

### Analysis of small RNA reads

#### Mapping

The adaptor sequence, AGATCGGAAGAGCACACGTCTGAACTCCAGTCAC, was trimmed from raw reads with cutadapt v.2.4 (65). Reads were mapped with STAR_2.6.0a (66) to TAIR10 with the following parameters: *outFilterMultimapNmax 20, alignIntronMax 1, outFilterMismatchNmax 1, outFilterMismatchNoverLmax 0, outFilterScoreMinOverLread 0, outFilterMatchNminOverLread 0, outFilterMatchNmin 15, alignSJDBoverhangMin 100*. No rRNA or tRNA reads were removed prior to mapping. Mapped reads were quantified with featureCounts from subread-1.6.3 (67) with an Araport11 transcriptome reference file custom modified to also include intergenic regions.

#### Initial quality controls

Reads were not quality controlled before mapping as FastQC on small reads is not recommended. On the other hand, the samples were validated based on principal component analyses and distance plot matrices prior to the differential expression analysis. Both PCA and distance plots were based on vst-transformed normalized reads (RPM) (68). One of the *ski2-5* replicates in experiment A was removed from further downstream analysis as it was a clear outlier in the PCA and distance plots (Supplementary Figure S1A,B). Using same argument, the sole biological replicate of *pel1-1* in experiment B was included for further inspection of differentially expressed siRNA populations as it clusters close to all three replicates of *pel1-2* (Supplementary Figure S1C,D). Results from the sole replicates of *ski2-5* (Experiment A) and *pel1-1* (Experiment B) did not enter formal statistical analysis via DEseq2 as explained below, but were exploited to show siRNA accumulation at selected loci of interest emerging from other analyses. For *pel1-1*, the loci of interest were found by rigorous analysis of siRNA data from wild-type and *pel1-2*, while for *ski2-5* (Experiment A), the loci of interest were found by analysis of siRNA data from wild-type, *ski3* and *rrp45b* (Experiment A) and wild-type and *ski2-5* in Experiments B, C (Supplementary Figure S2A,B). The data resulting from small RNA sequencing of libraries from experiment C was validated prior to differential expression analysis in the same manner, but no sample outliers were found (Supplementary Figure S1E,F).

#### Differential expression analysis

The package DESeq2 (68) was used for the differential expression analysis. Normalized reads were estimated for size factors (69) and a generalized linear model was used. The DESeq2 results were extracted with manual contrasting of mutant versus WT. A gene was considered to have differentially expressed small RNA levels compared to WT if the *P*_adj_ < 0.05 as assessed by Wald’s tests. Fisher tests were used to assess significance of enrichments of miRNA targets in sets of genes producing differentially expressed siRNAs. All genes with differentially expressed siRNAs from the three DESeq2 analyses are listed in the supplemental tables (Supplementary Tables S2–S4). Genes were categorized as known miRNA targets based on a list of experimentally validated targets (Supplementary Table S5).

#### Calculating absolute distances of small RNA reads to the cleavage sites

Genomic ranges of known miRNA targets were extracted from BigWig files and bw counts on each genomic position were normalized to the total amount of mapped reads and a mean was calculated by accounting for sample replicates. The genomic positions of a miRNA target and the 1 bp genomic position of its corresponding cleavage site were converted to transcriptomic positions using the ensembldb package (70).

#### Analysis of phasing

The first nucleotide adjacent to a known cleavage site (CS) of a miRNA target was designated as nucleotide (nt) 1 on both 5′ and 3′ CFs. Afterwards, the CFs were split into 21-nt intervals (Figure 6A). An siRNA was counted to belong to phase 1 if it mapped to the target transcript in any 21-nt interval arising from the CF being split from nt position 1 (e.g. 1-21, 22-42, 43-36 etc.). Only perfectly matching 21-nt long reads (cigar string = 21M) were included in the phasing analysis. The siRNAs were considered to be phased if the majority of reads fall into phase 1. The 2 nt displacement of the minus strand was not taken into consideration in the code and therefore a clear division of reads mapping to either the minus or plus strand is visible in phase 1 and 2 for the Tas1C example (Figure 6B).

#### Analysis of trimming and tailing

The trimming and tailing analysis of sRNA reads mapping to the extremity of the 5′ CF in *PHB* and *TCP2* was performed by filtering the BAM files on an approximately 10 nt match in sequence—10 nt from the cleavage site. Whether a read was trimmed or tailed and how many nucleotides were missing from or added to the end of the original CF end was computed on the basis of the sequence and cigar string.

#### Analysis of the relationship between miRNA:mRNA binding affinity, miRNA expression and propensity to trigger siRNA production

We used a dataset consisting of the 69 miRNAs with unique mature sequence paired with their 151 targets from Supplementary Table S5. These sets of miRNAs and target mRNAs gave rise to a total of 287 miRNA:target combinations. For each combination, the Gibbs free energy for the binding of the seed region of the miRNA (2–9 nt from 5′) to its corresponding target sequence was calculated using the R package Rmelting with default settings (https://aravind-j.github.io/rmelting/).

However, the Rmelting software does not have any algorithm to deal with mismatches at end positions and therefore for 14 pairs the Gibbs free energy was estimated using the RNAhybrid software (71). The miRNA:target sequences and the corresponding Gibbs free energies can be found in Supplementary Table S6. Estimation of Gibbs free energies for RNA hybridization involving an AGO-bound RNA strand is inaccurate with these tools because of the helical conformation of seed nucleotides even prior to hybridization (72). It is, therefore, an underlying assumption in the analysis that the error introduced by ignoring this reduced entropy loss upon hybridization is similar for all miRNA:target hybridization, such that the relative affinities are well approximated by the estimated Gibbs free energies.

To analyze if combined miRNA expression and miRNA:mRNA target affinity is related to the observed pattern of siRNA-producing miRNA targets in the *rrp4* mutant, we used logistic regression models. Only targets matched by miRNAs with unique seed sequence were included and the expression levels of such miRNAs were summed. This dataset consists of 153 miRNA:target combinations of which 30 produce significantly more siRNA in *rrp4* compared to WT. We fitted a binomial logistic regression model to see the effect of miRNA expression level on the probability of targets producing siRNA. To include the effect of Gibbs free energy of the miRNA(seed):target mRNA association, we fitted a saturated model. This model showed a significant covariance of miRNA expression level and binding energy (*P* = 0.047), such that the higher the miRNA is expressed the higher affinity it has to its target. We therefore reduced the model to see the effect of miRNA expression level and binding energy as covariates on the probability of targets producing siRNA. All logit models were validated with a Kolmogorov-Smirnov test (*P* > 0.05). To assess the effect of Gibbs free energy of the miRNA(seed):mRNA target association on siRNA production from different targets of the same miRNA, we focused on a subset of miRNAs that both have targets that produce siRNAs and targets that do not. Since many miRNA isoforms share targets, we also grouped the miRNAs into families (all isoforms and miRNA156 and 157 grouped), resulting in a data set of 9 miRNA families with 144 targets for which the status of siRNA production (y/n) was known. We fitted a linear mixed model with miRNA family as random effect to this data set.

All data analysis of the mapped and quantified reads were performed in R version 4.0.3, (https://www.r-project.org/) and plots were generated with ggplot2 (https://ggplot2.tidyverse.org/). All R code is available at the following Github repository: https://github.com/MariaLouisaVigh/ExosomePelota.

## RESULTS

### Inactivation of the exosome subunit RRP45B results in accumulation of RISC 5′-cleavage fragments

**Figure 1.**
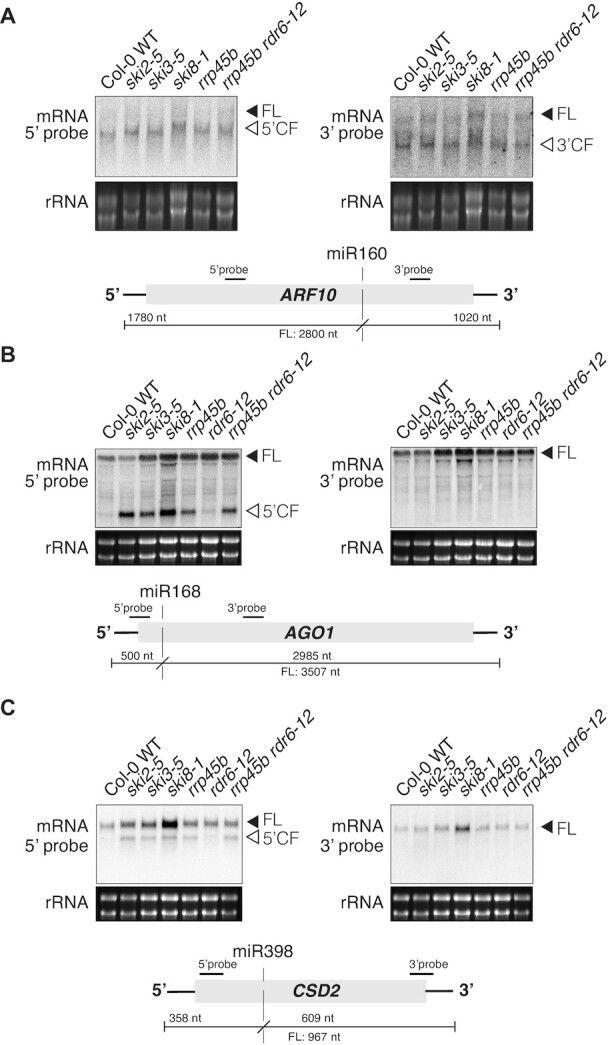
Accumulation of 5′ cleavage fragments in *rrp45b*. (**A**) Northern blot analyses of 20 μg total RNA from inflorescences. Radiolabeled probes specific to either the 5′ or the 3′ cleavage fragment of ARF10 were hybridized consecutively to the same membrane. Arrows indicate the full length (FL) and the 5′ or 3′ cleavage fragment (CF). Localization of the probes relative to the miR160 cleavage site (vertical dashed line) and expected cleavage fragment sizes are illustrated below the blots. (**B**) Same analysis as in (A) carried out with the miR168 target AGO1. (**C**) Same analysis as in (A) carried out with the miR398 target CSD2.

To answer the question of whether the RNA exosome mediates the degradation of RISC 5′-cleavage fragments that we previously showed to depend on the components of the SKI complex, SKI2, SKI3, SKI8, we performed northern blot analysis of RNA prepared from *rrp45b* mutants (also known as *cer7-3*). *RRP45B* is one of two genes encoding the core exosome subunit RRP45 in Arabidopsis, and in contrast to core exosome subunits encoded by single genes, knockouts of both *RRP45B* and *RRP45A* are viable (60,63). *RRP45B* was chosen for initial analyses, because its inactivation leads to production of ectopic siRNAs from some mRNAs (41). Our analyses showed that the *rrp45b* mutant exhibits overaccumulation of several miRNA-guided 5′-cleavage fragments (Figure 1A–C). Such overaccumulation may be subtle (ARF10/miR160, Figure 1A) or clear (AGO1/miR168, CSD2/miR398; Figure 1B,C), but is in all cases near-identical to what is observed in *ski2*, *ski3* and *ski8* mutants. The higher level of full length CSD2 mRNA detected specifically in *ski8* mutants may be due to the fact that the SKI8 protein has functions in complexes other than the cytoplasmic SKI complex, including the RNA polymerase II-associated Paf1c implicated in pre-mRNA synthesis and processing (73–75). In addition, as seen previously for *ski2-4* mutants (42), mutation of *RDR6* did not affect the level of 5′-cleavage fragments detected in *rrp45b* mutants (Figure 1A–C). We conclude from these observations that the degradation of several RISC 5′-cleavage fragments proceeds via a canonical SKI2-3-8-exosome dependent pathway.

### *rrp45b* and *ski3* mutants exhibit defective limitation of secondary siRNAs similar to *ski2*

**Figure 2.**
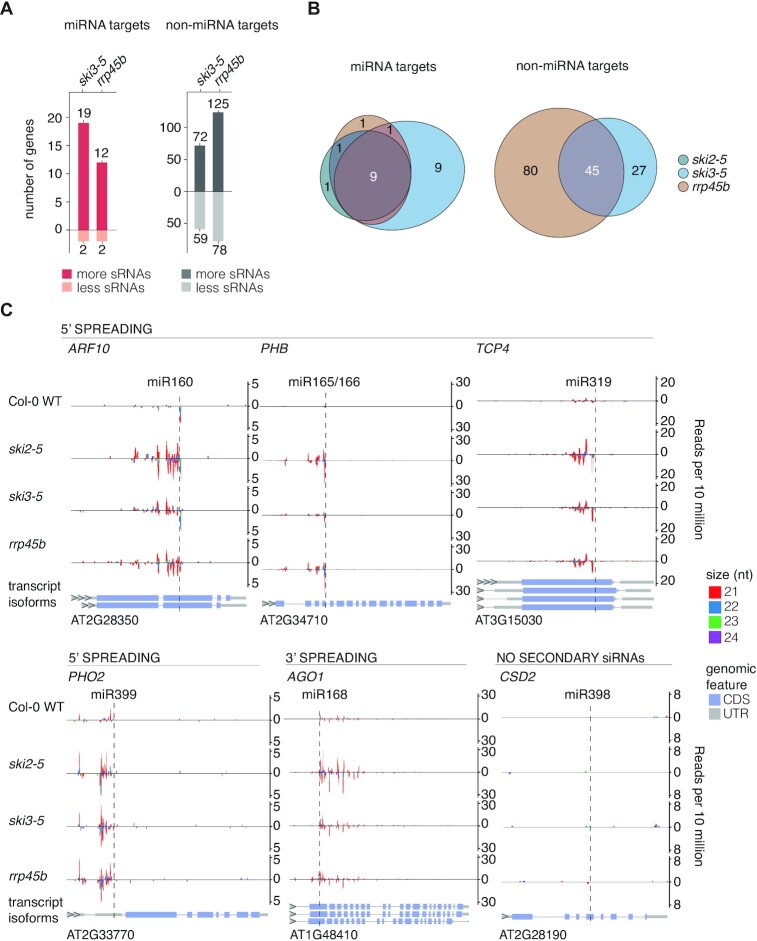
SKI3 and RRP45B limit secondary siRNA production on miRNA targets. (**A**) Bar plots depicting number of known miRNA targets (red bars) and number of mRNAs not known to be miRNA targets (non-miRNA targets, gray bars) which produce either more or less secondary siRNAs in *ski3-5* or *rrp45b* compared to Col-0 WT (Wald test, *P* < 0.05). The enrichment of miRNA targets in genes producing more siRNAs are highly significant in *ski3-5* and *rrp45b* (Fisher-test for *ski3-5*: *P* < 2.2 × 10^−16^, Fisher-test for *rrp45b*: *P* = 1.39 × 10^−15^). (**B**) Euler diagram of the overlap in known miRNA targets (left) and the overlap in non-miRNA targets (right) with ectopic siRNA accumulation in *ski2-5, ski3-5* and *rrp45b*. (**C**) Examples of siRNA accumulation on endogenous miRNA targets. Four examples with siRNAs mapping to the 5′ cleavage fragment (*ARF10, PHB, TCP4* and *PHO2*), one example of siRNAs mapping to the 3′ cleavage fragment (*AGO1*), and one example of no siRNA accumulation despite detection of a stable 5′ cleavage fragment (*CSD2*) are shown. The *y*-axis shows the number of small RNA reads per 10 million. Positive read counts indicate sense orientation, negative read counts indicate antisense orientation. The *x*-axis is the TAIR10 genomic coordinates of the target. sRNAs of sizes 21–24-nt are plotted and the sizes are distinguished with colors as indicated. Vertical dashed lines indicate miRNA-guided cleavage sites. The different transcript isoforms are illustrated for each gene with 5′- and 3′-UTRs in grey, exons in blue and introns as connecting lines. All miRNA targets are plotted in the 5′ to 3′ reading direction and their gene identifiers are noted below the transcript isoforms. Small RNA libraries prepared from two biological replicates were used for all analyses except for *ski2-*5 for which a single replicate had to be used, see Materials and Methods and Supplementary Figure S1A,B.

We next tested whether RRP45B and SKI3 are also necessary for avoidance of miRNA-induced secondary siRNA production, similar to SKI2. Small RNA-seq with RNA extracted from *ski3* and *rrp45b* mutants showed that miRNA targets were enriched among mRNAs producing ectopic siRNAs (Figure 2A), similar to what we reported previously for *ski2-4* (42). Comparison to a set of miRNA targets found repeatedly in multiple small RNA-seq experiments conducted with *ski2-5* and *ski2-4* to produce increased levels of siRNAs (Supplementary Figure S2, see Materials and Methods) showed that very similar sets of miRNA targets produced secondary siRNAs in *ski2*, *ski3* and *rrp45b* mutants (Figure 2B). Moreover, ectopic siRNAs mapped close to miRNA-guided cleavage sites (Figure 2C), as described previously for *ski2-4* mutants (42). The abundance of the trigger miRNAs themselves was unchanged compared to wild-type in all of the mutants (Supplementary Figure S3A), excluding increased trigger miRNA levels as a possible explanation for the increased siRNA production. We also verified that for all three mutants, no secondary siRNAs mapping to CSD2 were detectable (Figure 2C), despite the clear stabilization of CSD2 5′-cleavage fragments (Figure 1C).

Since knockout mutants in genes encoding two distinct components of the SKI2-3-8 complex, an ATP-binding site mutant of *SKI2* (*ski2-4*) (42), and a mutant in *RRP45B* give highly similar profiles of illicit secondary siRNAs on miRNA targets, and lead to similar stabilization of 5′-cleavage fragments, we conclude that SKI2-mediated exosome function, presumably 3′-5′ exonucleolysis of 5′-cleavage fragments, underlies its role in limitation of secondary siRNA production. We stress that this is a not a trivial result, because the free, stable 5′-cleavage fragment cannot be the direct template for RDR6 in those cases in which secondary siRNAs map to the 3′-cleavage fragment. In addition, the 5′-cleavage fragment released from RISC is generally unlikely to serve as an RDR6 template because of the existence of examples such as CSD2 for which stable 5′-cleavage fragments do not give rise to siRNA populations, or ARF10 for which stable 5′-cleavage fragments are only slightly more abundant in mutants in components of SKI or exosome complexes than in wild-type, yet miRNA-triggered siRNA production is robust. Thus, exosomal degradation of 5′-cleavage fragments is required to avoid siRNA production and spreading in both 5′- and 3′-directions relative to the miRNA-guided cleavage site, a point that will be treated in depth in the discussion. Consistent with our results, miRNA targets have previously been observed to be represented among siRNA-generating transcripts in mutants of the exosome or of the RST1/RIPR complex implicated in connecting cytoplasmic exosome and SKI2-3-8 complexes (36,38).

### miRNA-induced secondary siRNA production upon inactivation of *PEL1*

**Figure 3.**
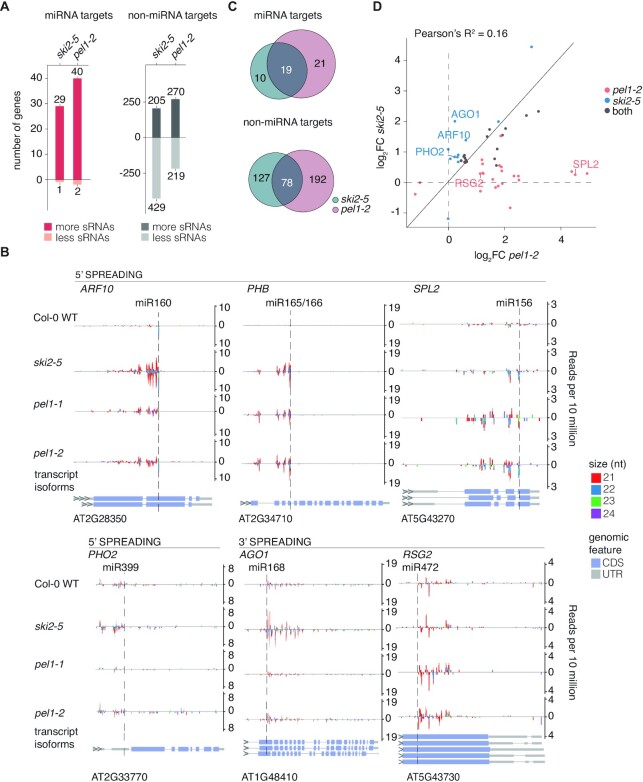
PEL1 limits secondary siRNA amplification from miRNA targets. (**A**) Bar plots depicting number of known miRNA targets (red bars) and number of mRNAs not known to be miRNA targets (non-miRNA targets, gray bars) which produce either more or less secondary siRNAs in *ski2-5* or *pel1-2* compared to Col-0 WT (Wald test, *P* < 0.05). The enrichment of miRNA targets in genes producing more siRNAs is highly significant in *ski2-5* and *pel1-2*, (*P* < 2.2 × 10^−16^ in both cases, Fisher-test). (**B**) Examples of siRNA accumulation on endogenous miRNA targets, organized as in Figure 2C. Four examples with siRNA accumulation on the 5′ CF (*ARF10, PHB, PHO2* and *SPL2*), and two examples of accumulation on the 3′ CF (*AGO1* and *RSG2*) are shown. (**C**) Euler diagrams showing the overlaps in known miRNA targets (top) and in non-miRNA targets (bottom) with ectopic siRNA accumulation in *ski2-5* and *pel1-2*. The number of miRNA targets in the intersection between *ski2-5* and *pel1-2* is significant (Fisher-test: *P* = 0.02905). (**D**) Scatter plot of log_2_ of the fold change of read densities (RPM(mutant)/RPM(WT)) of siRNAs (log_2_FC) mapped to miRNA targets in *pel1-2* (*x*-axis) and *ski2-5* (*y*-axis). Only miRNA targets with significantly higher siRNA read counts in either mutant compared to Col-0 are included. miRNA targets shown in Figure 3B are indicated. miRNA targets are colored according to whether they produce more siRNAs than wild-type in only *pel1-2* (red), only in *ski2-5* (blue) or in both mutants (gray). Small RNA libraries prepared from biological triplicates were used for all analyses presented in this figure (except for *pel1-1*, which is a sample without a replicate (Supplementary Figure S1C,D).

We next analyzed the effect of inactivation of the gene encoding PEL1. Metazoan Pelota is necessary for subunit dissociation of stalled ribosomes with an empty A-site, for example in the NSD pathway that eliminates faulty mRNAs without a stop codon (46). Consistent with this biochemical role, *pel1* mutants accumulate 5′-cleavage fragments generated by some miRNA-guided RISCs with targets in open reading frames, but not in 3′-UTRs (54). We found that the *pel1-2* mutant produced siRNAs from a limited set of mRNAs significantly enriched in known miRNA targets (Figure 3A), similar to *ski2-5*. siRNA production from most of these targets was also detected in the independent *pel1-1* mutant (Supplementary Figure S4A). The amplified siRNAs mapped adjacent to miRNA target sites in individual target mRNAs (Figure 3B; Supplementary Figure S4B), consistent with a miRNA-RISC-triggered process. Importantly, the abundances of the trigger miRNAs themselves were unchanged compared to wild-type in all of the mutants (Supplementary Figure S3B), arguing against a trivial explanation of increased RISC activity as the cause of secondary siRNA production. Furthermore, the overlap in siRNA-producing miRNA-targets between *pel1-2* and *ski2-5* was significant, in contrast to the overlap in non-miRNA targets (Figure 3C). Nonetheless, around half of the miRNA-targets found to produce increased siRNA levels in *pel1-2* did not do so in *ski2-5*. The opposite statement was also true, as roughly one-third of the siRNA-overproducing miRNA targets found in *ski2-5* did not produce increased levels of siRNAs in *pel1-2* (Figure 3C). More generally, increased siRNA levels from miRNA targets in *ski2-5* were not correlated with those in *pel1* mutants (Figure 3D; Supplementary Figure S4C), an important point that is well illustrated by inspection of individual examples. *PHO2/UBC24* targeted by miR399 is an expected example of increased secondary siRNA production specifically in *ski2-5*, but not in *pel1* mutants (Figure 3B,D), because the miR399 target sites are located in the 5′-UTR (Figure 3B), where 80S ribosomes required for recognition by PEL1 are not assembled. However, even for targets with miRNA sites in open reading frames, siRNA overproduction specific to *ski2-5* could be observed, as illustrated clearly by the examples miR168-*AGO1* and also to some extent miR160-*ARF10* (Figure 3B,D). *AGO1* mRNA consistently gives rise to much higher siRNA levels mapping to the 3′-cleavage fragment in mutants of the exosome and SKI complexes (Figure 2C), but not in any of the two independent *pel1* mutants (Figure 3B; Supplementary Figure S4C). Similarly, the increase in siRNAs for ARF10 in *pel1* mutants was much smaller than that observed in *ski2* mutants (Figure 3B,D). Conversely, miR156-*SPL2* gives rise to secondary siRNAs mapping to the 5′-cleavage fragment of SPL2 mRNA only in *pel1* mutants, and miR472 triggers more robust increases in siRNAs mapping to the 3′-cleavage fragment of *RSG2* mRNA in *pel1* than in *ski2-5* mutants (Figure 3B,D; Supplementary Figure S4C). These observations show that loss of PEL1 function leads to illicit secondary siRNA production from miRNA targets, similar, but not identical, to the consequence of inactivation of SKI2-3-8 and exosome complexes, indicating that PEL1 and SKI-exosome act to avoid miRNA-triggered siRNA production via different mechanisms. We exclude the trivial possibility of differential expression of miRNA targets between *ski2* and *pel1* mutants as the cause of differential siRNA production. Although small differences in expression levels of PHO2, AGO1 and SPL2 mRNAs were detected between *ski2* and *pel1*, there was no tendency for the target mRNA to be either up- or down-regulated in the genetic background in which siRNAs were produced (Supplementary Figure S4D–F). Clearly, it follows from the conclusion that SKI2 and PEL1 limit miRNA-induced siRNA production by different mechanisms that simultaneous inactivation of *SKI2* and *PEL1* should result in exacerbated siRNA production from miRNA targets with siRNAs in both single mutants. We could not verify this prediction, because *ski2*/*pel1* double mutants were embryonically lethal (Supplementary Figure S5).

### The nuclear exosome broadly suppresses siRNA production

**Figure 4.**
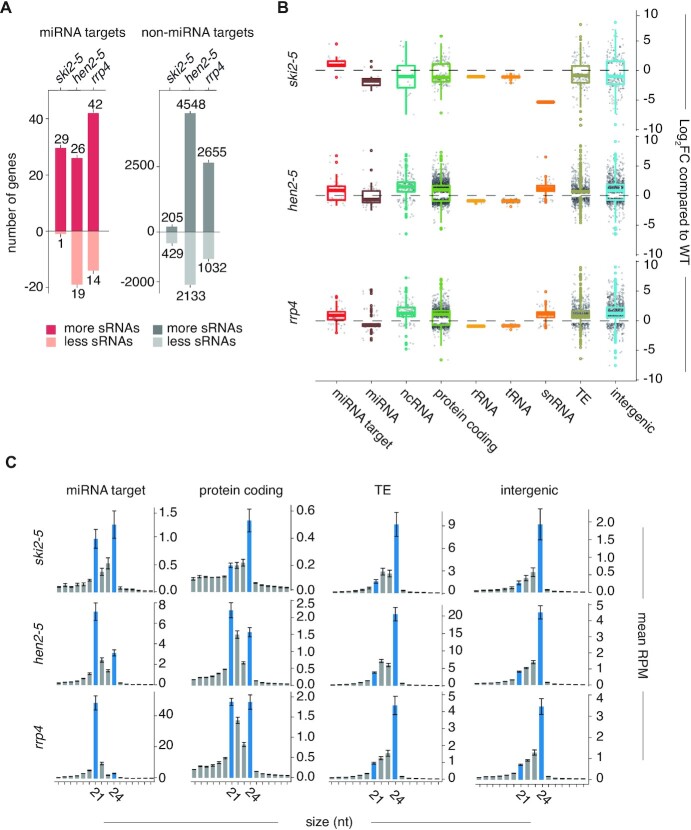
HEN2 and RRP4 have effects on siRNA production from RNAs beyond miRNA targets. (**A**) Bar plots depicting number of known miRNA targets (red bars) or number of non-miRNA targets (grey bars) which produce either more or less secondary siRNAs in *ski2-5, hen2-5* or *rrp4* compared to Col-0 WT (Wald test, *P* < 0.05). The enrichment of miRNA targets in genes producing more siRNAs is significant in *ski2-5* and *rrp4* (Fisher-test for *ski2-5*: *P* < 2.2 × 10^−16^, Fisher-test for *rrp4*: *P* = 1.7 × 10^−4^). The proportion of miRNA targets found in genes with lower levels of siRNAs in the *ski2-5* and *rrp4* mutants compared to WT is not significant (Fisher-test for *ski2-5*: *P* = 1.00, Fisher-test for *rrp4: P* = 0.07551). The enrichment of miRNA targets in genes producing both more and less siRNAs in *hen2-5* compared to WT is not significant (Fisher-test for miRNA target enrichment in genes with more siRNAs: *P* = 0.12, Fisher-test for miRNA target enrichment in genes with less siRNAs: *P* = 0.62). (**B**) Boxplot of all genes with significantly differentially expressed siRNAs mapped to them (*P*_adj._ < 0.05). The *y*-axis shows the log_2_ of the fold change of read densities (RPM(mutant)/RPM(WT)) of siRNAs mapped to genes. Genes are split into different types according to Araport11 annotations (GFF3 file custom modified to include annotations of intergenic regions). (**C**) Bar plots showing the size distribution of siRNAs mapped to miRNA targets, protein coding genes, transposable elements (TE) and intergenic regions in the different mutants indicated. Error bars show the standard error of the mean RPM in biological triplicates. Small RNA libraries prepared from biological triplicates were used for all analyses presented in this figure.

Given the importance of cytoplasmic exosome-dependent RNA decay systems for the avoidance of miRNA-triggered siRNA production, we next asked whether nuclear exosome-dependent RNA decay may also play a role. We initially analyzed mutants in two components: Knockout alleles of *HEN2* which encodes a nucleoplasmic relative of SKI2 (59), and a hypomorphic allele of *RRP4* encoding a core exosome component (61), expected to affect both nuclear and cytoplasmic exosome activities (76). Small RNA-sequencing demonstrated that although some miRNA targets did produce siRNA quantities different from wild-type (Figure 4A), the *hen2* and *rrp4* mutants exhibited vast differences in their small RNA profiles compared to wild-type and *ski2* (Figure 4B). In addition to the previously observed siRNA generation from divergent non-coding transcripts (76), many different classes of RNA, in particular mRNA, gave rise to small RNAs (Figure 4B). These ectopic small RNAs showed a size distribution typical of siRNAs resulting from DICER-LIKE cleavages with peaks at 21 and 24 nt (Figure 4C), indicating that they are *bona fide* siRNAs and not simply random degradation fragments. In addition, the relative accumulation of ectopic 24- and 21-nt siRNA species was, broadly speaking, consistent with the type of source transcript. 24-nt siRNAs dominated from intergenic transcripts and transposable elements while 21-nt siRNAs were more abundant from mRNAs (Figure 4C). Because of the many mRNAs present in the siRNA-producing set, we also tested whether the Zn-finger protein SOP1, related to the key component of the mammalian nuclear Poly(A)-directed exosome targeting complex, ZCCHC1 (77), showed similar deregulated siRNA production. Unlike *hen2* and *rrp4* mutants, however, *sop1-5* did not exhibit substantial ectopic siRNA production (Supplementary Figure S6A), precluding SOP1-dependent nuclear (pre)-mRNA decay as the major pathway required to limit massive mRNA-derived siRNAs observed in *hen2* and *rrp4*.

### *RRP4* has a more profound effect on limitation of miRNA-induced secondary siRNAs than either *SKI2* or *HEN2*

**Figure 5.**
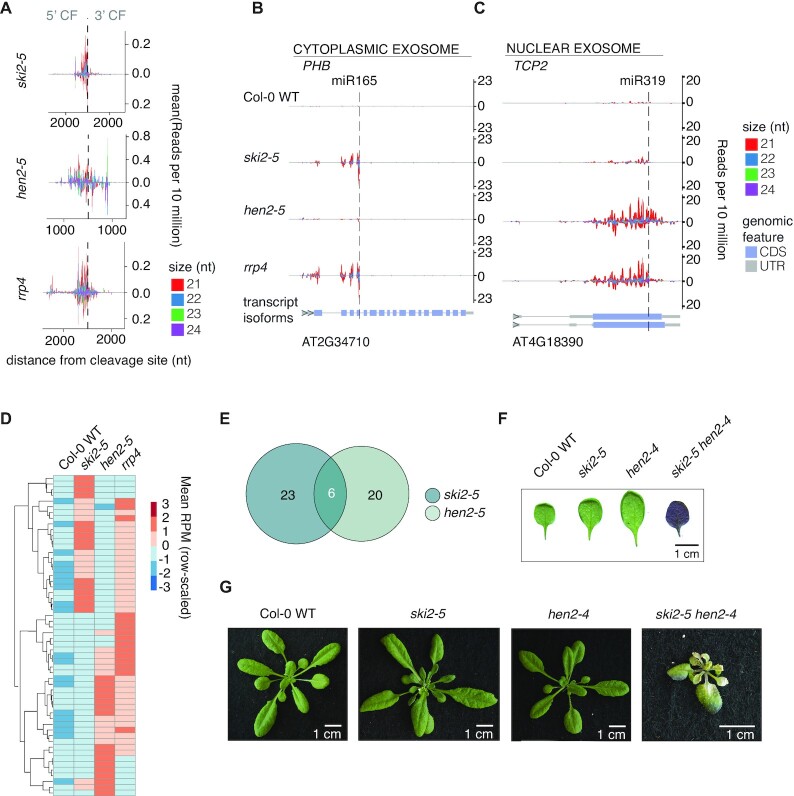
Distinct patterns of miRNA-induced secondary siRNA production in cytoplasmic and nuclear exosome mutants. (**A**) Metaplot of siRNA read densities (RP10M) along miRNA target transcripts with significantly higher siRNA production in mutants than in wild-type. Position 0 is defined by miRNA-guided cleavage sites. (**B**) An example of ectopic secondary siRNA accumulation on a known miRNA target, *PHB*, in *ski2-5* and *rrp4* mutants, but not in *hen2-5*. (**C**) An example of ectopic secondary siRNA accumulation from a known miRNA target, *TCP2*, in *hen2-5* and *rrp4* mutants, but not in *ski2-5*. Plots are organized as in Figures 2C and 3B. (**D**) Heatmap of known miRNA targets with significantly more siRNAs produced in *ski2-5, hen2-5* and *rrp4* compared to WT (*P*_adj._ < 0.05). In the heatmap, the *z*-score of the mean RPM of siRNAs mapped to each miRNA target in WT, *ski2-5, hen2-5* and *rrp4* are used. The heatmap is clustered by targets. Small RNA libraries prepared from biological triplicates were used for all analyses presented in this figure. (**E**) Euler diagrams showing overlap in siRNA-producing miRNA targets between *ski2-5* and *hen2-5* mutants. (**F**) Abaxial side of detached first true leaves of Col-0, *ski2-5*, *hen2-4* and *hen2-4/ski2-5*. (**G**) Rosette phenotypes of Col-0, *ski2-5*, *hen2-4* and *hen2-4/ski2-5* mutants. The plants were photographed after 25 days of growth.

The fact that a high number of mRNAs gives rise to secondary siRNA production in *hen2* and *rrp4* mutants might mean that miRNA targets are included in the set of ectopic siRNA producers by coincidence. If so, the production of siRNAs from these targets would not allow inferences on the possible implication of HEN2 and RRP4 in limitation of miRNA-induced siRNA production. We therefore first inspected the pattern of accumulation of siRNAs mapping to miRNA targets in *hen2* and *rrp4*. Metaplots of siRNA read densities centered on miRNA-guided cleavage sites showed an asymmetry with higher read counts 5′ to cleavage sites and lower read counts 3′ to cleavage sites (Figure 5A). This pattern is indicative of a miRNA-RISC-triggered process and is consistent with the fact that most miRNA-triggered secondary siRNAs map 5′ to the cleavage site, perhaps as a consequence of asymmetry in base pairing strength between the 5′ (seed) and 3′ halves of miRNAs to their targets (42). The analysis of siRNAs mapping to miRNA targets in *hen2* and *rrp4* also showed that while siRNA abundances on individual targets tended to be highest close to the miRNA target site, they covered larger parts of target transcripts compared to what is observed in *ski2*, *pel1* and *rrp45b*, and were generally not restricted to either 5′- or 3′-cleavage fragments (Figure 5A–C). We next compared the identities of miRNA target mRNAs giving rise to secondary siRNAs in *ski2*, *hen2* and *rrp4*. These analyses showed that nearly perfectly reciprocal sets of miRNA targets gave rise to secondary siRNAs in *ski2* and *hen2*, and that the union of those two sets largely matched the set of miRNA targets giving rise to siRNAs in *rrp4* (Figure 5D,E; Supplementary Figure S7A,B). We did not include *hen2/ski2* double mutants in this analysis, because their strong developmental phenotype (Figure 5F,G) made it impossible to produce comparable inflorescence tissues for siRNA analysis. A tempting and straightforward interpretation of the observation of reciprocity in miRNA-target/mRNA pairs giving rise to secondary siRNAs between *ski2* and *hen2* mutants is that SKI2 and HEN2 perform biochemically similar functions in limiting miRNA-induced secondary siRNA, with SKI2 acting in the cytoplasm (57), and HEN2 acting in the nucleoplasm (60). Clearly, this interpretation would mean that some miRNA-mRNA targeting events occur preferentially in the cytoplasm while others occur preferentially in the nucleus. If so, the tendency of individual miRNA-targets to produce siRNAs mapping both 5′ and 3′ to miRNA target sites in *hen2* mutants may be explained by a higher tendency of nuclear RDR6 to engage in amplification, including 3′-spreading initiated by a subset of secondary siRNAs. More trivial explanations than nuclear-cytoplasmic partitioning of miRNA-mRNA targeting events are also possible, however. For example, inactivation of HEN2/RRP4 may allow nuclear escape of defective mRNA species and hence provide a pool of mRNA particularly sensitive to RdRP recruitment upon RISC targeting in the cytoplasm. Finally, we note that the similar profiles of ectopic siRNA production between *ski2* and *rrp45b*, but the very different effects observed in *rrp45b* on the one hand and *hen2* and *rrp4-2* on the other, suggest that inactivation of *RRP45B* may mostly affect cytoplasmic exosome activity. A simple separation of nuclear and cytoplasmic exosome activities between RRP45A and RRP45B does not seem to operate, however, because contrary to *hen2* mutants, *rrp45a* mutants did not produce ectopic siRNAs from miRNA targets (Supplementary Figure S6B).

### Illicit secondary siRNAs are not phased

**Figure 6.**
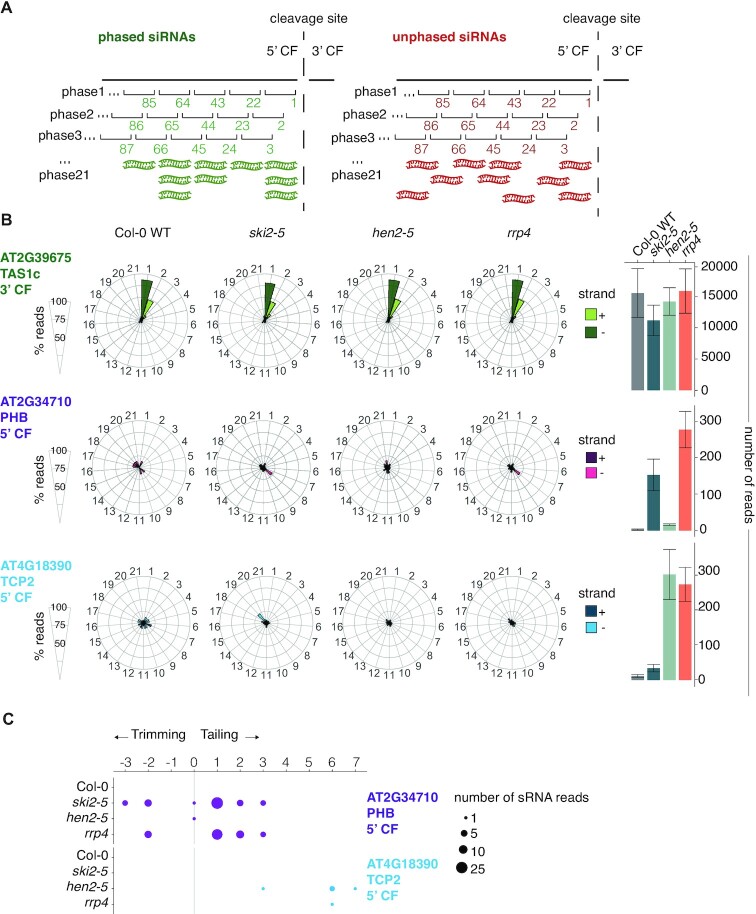
Secondary siRNAs amplified from endogenous miRNA targets in *ski2-5, hen2-4* and *rrp4* mutants are not phased. (**A**) Illustration of phased and unphased siRNA populations, and definitions of the phase registers used for the analysis in (B). (**B**) Distribution of siRNAs mapping to *TAS1c*, *PHB* and *TCP2* in the 21 possible phases in *Col-0*, *ski2-5*, *hen2-5* and *rrp4* mutants. *TAS1c* serves as a control known to generate phased siRNA populations. Dark shades, reads mapping to the plus strand; light shades, reads mapping to the minus strand. Bar plots on the right show the total number of reads underlying the analysis of phase distributions in each genotype. Error bars indicate the standard error of the read count biological triplicates. (**C**) Dot plot representation of heterogenic 5′ ends of the small RNA reads which map to the extremity of the *PHB* and *TCP2* 5′ CF in WT, *ski2-5, hen2-5* and *rrp4*. The circle size represents the number of sRNA reads with a certain number of tailed/trimmed nucleotides found to map to the 5′ CF extremity. Small RNA libraries prepared from biological triplicates were used for all analyses presented in this figure.

We next analyzed the miRNA-induced siRNA populations in *ski2*, *hen2* and *rrp4* for phasing relative to the cleavage site, as this may reveal important insight into their mechanism of generation. A phased pattern of accumulation implies that secondary siRNAs resulting from the initial miRNA-dependent recruitment of RDR6 do not trigger further amplification, because small RNA-guided cleavage by AGO1 occurs opposite of nucleotides 10–11 of the guide RNA, thus leading to a 10-nt phase shift if re-amplification occurs (Figure 6A). In contrast, multiple scenarios can explain lack of phasing, including reiterative amplification initially starting from a well-defined point, and single-round amplifications starting from template RNAs not perfectly in phase. We detected no phasing of the illicit siRNA populations mapping to PHB and TCP2, in contrast to the phased *TAS1c* siRNAs (Figure 6B). At least part of the reason for this result appears to be unaligned 3′-ends of the 5′-cleavage fragments: for PHB, we found that the 3′-most siRNA species, resulting from the first Dicer-catalyzed cleavage event, occurred in at least 6 different phases because of nucleotide tailing and trimming of the PHB 5′-cleavage fragment (Figure 6C).

### Illicit siRNA production correlates with miRNA abundance and miRNA:target mRNA affinity

**Figure 7.**
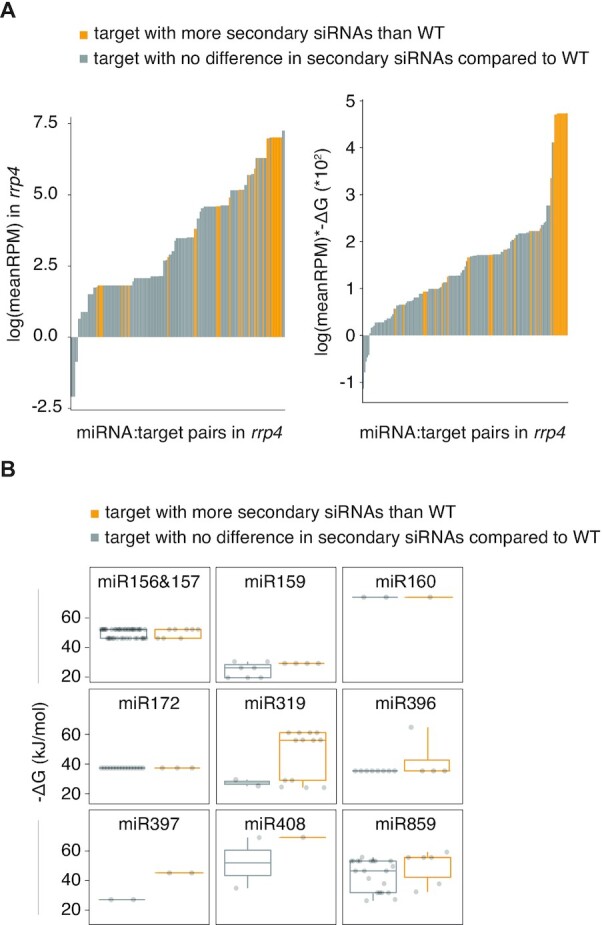
Correlation between miRNA expression level, binding energy and miRNA-induced secondary siRNA production. (**A**) The left bar plot shows miRNA:target pairs ranked by expression level (RPM) of the miRNA in *rrp4* and colored according to whether significantly higher siRNA counts are detected in *rrp4* than in wild-type. The log(meanRPM) of the miRNA gene is plotted on the *y*-axis for each miRNA:target pair on the *x*-axis (total number of miRNA:target pairs = 153). In a logit model there is a significant and positive effect of miRNA abundance on miRNA-induced siRNA production (LRT, *P* = 1.073 × 10^−5^). The right bar plot shows the same miRNA:target pairs, colored as in the left plot, but ranked instead by miRNA expression level (RPM) and binding energy (-Δ*G* (kJ/mol)) as a covariate. A logit model with the covariate as an effect of miRNA-induced secondary siRNA production was fitted and the effect is significant and positive (LRT, *P* = 5.789 × 10^−9^). The Gibbs free energy is calculated for the base pairing in the miRNA seed region (nt 2 to 9) to its target. Yellow bars, miRNA genes with a significantly higher number of siRNAs in *rrp4* compared to wild type; gray bars, miRNA genes with no significant difference in siRNAs in *rrp4* compared to wild -type. (**B**) Binding energy (Δ*G* (kJ/mol)) of seed region of miRNA to different targets of the same miRNA family. In total there were 144 miRNA:target combinations where members of the same miRNA family could pair to more than one target – some of which produce siRNAs (yellow) and others that do not (gray). In a linear mixed model with miRNA family as random effect, the binding is significantly stronger (lower Δ*G*) when secondary siRNAs are produced (*χ^2^*, *P* = 0.016). Small RNA libraries prepared from biological triplicates were used for all analyses presented in this figure.

This and our previous study (42) show that occurrence of stable 5′-cleavage fragments is not the trigger of production of secondary siRNAs from miRNA targets in *ski2, ski3* and *rrp45b* mutants. For example, *MYB33* (miR159) and *CSD2* (miR398) show stable 5′-cleavage fragments in *ski2* mutants, but no siRNA production, ARF10 (miR160) shows readily detectable 5′-cleavage fragments in both wild-type and *ski2* mutants, but siRNA production only in *ski2*, and *AGO1* (miR168) shows a stable 5′-cleavage fragment, but increased siRNA abundances mapping to the 3′-cleavage fragment in *ski2* mutants. With the extended set of miRNA targets found to produce secondary siRNAs in *rrp4* mutants, we therefore asked if other properties of miRNA/target pairs could be identified that correlate with the tendency to initiate secondary siRNAs. We previously proposed that RISC itself acts as a trigger for siRNA amplification, provided that its dwell time on target RNA exceeds a certain threshold that allows assembly of the amplification machinery around RISC (42). This model predicts that the probability of engaging in siRNA production should increase with the frequency of RISC:target mRNA encounters and with miRNA:target mRNA affinity. We therefore first tested if siRNA-producing miRNA:target pairs can be sorted from non-siRNA producing pairs based on miRNA abundance as revealed by small RNA-seq. By fitting a logistic regression model, we found that the relationship between siRNA production and trigger miRNA expression level was significant (LRT, *P* = 1.073 × 10^−5^, Figure 7A, left). We next tested if prediction of siRNA-production from miRNA:target pairs could be improved by taking miRNA:target affinity into account in addition to miRNA expression level. A saturated logistic model showed significant covariance of miRNA expression levels and miRNA:target affinity (LRT, *P* = 0.045). Therefore, we fitted a logistic regression model with the covariate term (miRNA abundance * miRNA:target affinity). This showed that both miRNA:target affinity and miRNA expression level are significant predictors of siRNA production (LRT, *P* = 5.789 × 10^−9^), such that the more abundant a miRNA is and the stronger it binds to its target, the more likely it is to induce siRNA production. Compared to the model with only miRNA abundance as fixed effect, the covariate model fitted the data better, as seen by the better separation of siRNA producers from non-siRNA-producers (Figure 7A, right). The better data fit and higher prediction power of the covariate model was also apparent from quantitative measures of fit (Akaike Information Criterion AIC = 122, Tjur’s R^2^ = 0.27 for the covariate model; AIC = 136, Tjur's R^2^ = 0.14 for the model with only miRNA expression as fixed effect). These results indicate that both miRNA expression level and miRNA:target mRNA affinity are relevant variables to determine whether siRNA production is triggered. Because miRNA:target mRNA affinity is more directly connected to the RISC dwell time hypothesis, we sought to further test its relevance. To this end, we selected 9 miRNA families for which both siRNA-producing and non-siRNA producing target pairs could be identified, and asked whether miRNA:target mRNA affinity could explain the different outcomes in terms of siRNA production within miRNA families. We found that the tendency for siRNA-producing miRNA:target pairs to be of higher affinity than non-siRNA-producing miRNA:target pairs was statistically significant (χ^2^ = 0.016, Figure 7B). These results corroborate the importance of miRNA:target mRNA affinity in determining whether siRNA amplification results from RISC:target encounters.

## DISCUSSION

### Exosomal degradation of the 5′-cleavage fragment as an accelerator of RISC dissociation

We previously used the term ‘RISC-trigger model’ for miRNA-triggered secondary siRNA production via RISC itself bound to cleaved target RNA as the key initiating element (42). In contrast, we refer to models that propose initiation of secondary siRNA production through target RNA cleavage fragments by virtue of their aberrant properties as the ‘aberrant RNA model’. The RISC-trigger model proposes as its central feature that the dwell time of RISC on target RNA is decisive for recruitment of the machinery required for secondary siRNA formation, such that long dwell times favor RdRP recruitment, while rapid RISC dissociation leads to target regulation in the absence of secondary siRNA formation. Evidence consistent with the RISC-trigger, but incompatible with the aberrant RNA model, includes examples of miRNA targets with stable 5′-cleavage fragments in *ski2* mutants that either fail to produce secondary siRNAs or produce secondary siRNAs mapping to the 3′-cleavage fragment. More direct support for the RISC-trigger model comes from the observation that miR173 triggers secondary siRNAs from *TAS1* and *TAS2* precursors in the complete absence of slicer activity of AGO1 such that no cleavage fragments are generated (11). Likewise, AGO7-miR390 is capable of triggering *TAS3* secondary siRNAs from uncleavable miR390 sites in *TAS3* precursor RNAs (78). In addition, recent recapitulation of AGO1-miR173- and AGO7-miR390-triggered tasiRNA generation in cell-free systems showed that RDR6 and AGO physically associate in RISC:target associations leading to tasiRNA production, thus verifying a central element of the RISC trigger model (79,80). In the following, we offer an interpretation of the results described here within the framework of the RISC-trigger model. We stress that despite the support outlined above, this model cannot at present be viewed as fully supported by evidence, and we take care to emphasize predictions that arise specifically from interpretations of the present results on the basis of the RISC-trigger model.

The fact that inactivation of the *SKI3* and *RRP45B* genes leads to illicit miRNA-triggered siRNA production very similar to what is observed in *ski2* mutants strongly suggests that there is no previously unrecognized biochemical activity of SKI2 specifically linked to limitation of secondary siRNA production: this effect of SKI2 must also be explained by facilitating exosome function, almost certainly the degradation of 5′-cleavage fragments. But how does failure to degrade 5′-cleavage fragments lead to ectopic siRNA generation 3′ to the cleavage site, as in the example of *AGO1* mRNA? We suggest that SKI2/3/8-exosome-mediated degradation of 5′-cleavage fragments happens *before*, not after, RISC has fully dissociated from its cleaved target RNA: dynamic ‘breathing’ of base pairs between the 5′-cleavage fragment and the RISC-bound miRNA may allow interaction with SKI2/3/8 while RISC and cleavage fragments are still held together. In this way, exosomal decay of 5′-cleavage fragments would accelerate dissociation of RISC from cleaved target RNAs, and hence maintain dwell times short enough to avoid recruitment of the machinery required for secondary siRNA production. Clearly, such a mechanism predicts physical proximity between SKI2-3-8/exosome and RISC, a property that has not yet been observed in plants, but for which there is precedent in other systems. For example, the *Neurospora crassa* Argonaute protein QDE-2 participates in biogenesis of miRNA-like small RNAs by association with longer precursors such that exosome-mediated trimming of QDE-2-bound small RNA precursors results in mature RISC containing a QDE-2-bound small RNA (81).

### miRNA:target mRNA affinity as a determinant of siRNA production

It is a significant finding of the present study that rather than cleavage fragment levels, miRNA levels and miRNA:target mRNA affinity are good predictors of miRNA-induced secondary siRNA production, tested here in the *rrp4* mutant that offers the largest number of cases to be studied. Since high miRNA:target affinity results in slow RISC dissociation (82), this result directly supports the idea that long dwell times of RISC on target RNA triggers siRNA amplification. The dependence on trigger miRNA levels constitutes less direct support for the RISC trigger model, but is consistent with it, simply because a higher RISC concentration increases the number of RISC:target encounters, hence increasing the chance that some encounters will be long-lived enough to trigger siRNA production. We note that in addition to providing support for the RISC-trigger model, the relevance of miRNA:mRNA target affinity for secondary siRNA production may provide a simple explanation for the tendency of 22-nt miRNAs to induce siRNA amplification, even in the presence of SKI2-3-8/exosome function: the additional base pair between the target 5′-cleavage fragment and the 3′-end of the miRNA may prolong the average dwell time of RISC sufficiently that at least some RISC:target mRNA encounters lead to recruitment of the siRNA amplification machinery. This interpretation would also explain the recent observation that a 1-nt insertion mutant in miR398b to make it 22-nt long does not induce secondary siRNAs (83). In the RISC-trigger model, the mutant 22-nt miR398b does not induce siRNA amplification, because the additional nucleotide in the miRNA does not result in formation of additional target base pairs, and hence does not lead to longer-lived miR398b-RISC:target interactions.

### Relevance of aberrant RNA in siRNA amplification

Although the evidence against RISC-generated cleavage fragments as triggers of siRNA production via RDR6 from this and previous work (11,42,78,84,85) is now compelling, aberrant RNA clearly can play a role in RDR-mediated siRNA amplification given the many reports of ectopic siRNA production in mutants in mRNA decay (33–41). How may these seemingly opposing pieces of evidence be reconciled? We previously proposed that aberrant RNA may be particularly relevant for allowing reiterative RISC-RDR6-mediated siRNA amplification through as yet ill-defined mechanisms. It is also possible, however, that some aberrant RNAs act as *bona fide* triggers of RNA silencing, as suggested for the L1 GUS transgenic system used to decipher large parts of the genetics of amplified RNA silencing in plants (86). For example, RDRs other than RDR6 may convert some aberrant RNAs into dsRNA leading to production of a small population of primary siRNAs that may be further amplified via the RISC-RDR6 module. The silencing of the wax biosynthesis gene *CER3* in *rrp45b* mutants is particularly instructive in this regard. *CER3* siRNA production in *rrp45b* depends not only fully on the well-described RISC-RDR6 module (AGO1, SGS3, SDE5, RDR6) but also on RDR1 (41,87). Thus, this unique case may be explained by successive actions of RDR1 and RDR6 in siRNA production such that RDR1 might mediate formation of low-abundant primary siRNAs using aberrant *CER3* RNA stabilized in the *rrp45b* mutant as template. Given that *Arabidopsis* also encodes the three neighboring, closely related and functionally uncharacterized RDR3-5 (AT1G19910, AT1G19920, AT1G19930), it is possible that RDR function in primary siRNA formation is widespread, but recalcitrant to genetic analysis, except in rare cases where a single RDR enzyme is uniquely responsible for this step. It is also noteworthy that silencing of *CER3* in *rrp45b* mutants is suppressed by inactivation of the SKI complex (88), a result seemingly in opposition to the enhancement of miRNA-triggered siRNA production in *ski2* and *ski3* reported here. If an aberrant *CER3* RNA population triggers siRNA production in *rrp45b* mutants, it is likely to be degraded via the action of SKI-exosome complexes in wild-type cells. In the absence of the exosome, binding of the SKI complex might stabilize this RNA population for long enough to be used as a template for an RDR, perhaps RDR1, while in the absence of both SKI and exosome activity, an alternative RNA degradation pathway may take over to preclude the entry of this RNA population into the RNA silencing pathway.

### Distinct roles of PEL1 and SKI2 in limiting secondary siRNA production

PEL1, SKI2-3-8 and the cytoplasmic exosome have a clear role in degradation of RISC 5′-cleavage fragments and other NSD substrates (42,54). Yet, as discussed at length above, the stable 5′-cleavage fragments are not direct RdRP substrates, suggesting that the requirement for PEL1 for avoidance of miRNA-triggered siRNAs may not be related to degradation of 5′-cleavage fragments, and, by consequence, that inactivation of PEL1 and SKI2-3-8-exosome leads to miRNA-triggered siRNA production for different reasons. Consistent with this idea, the set of miRNA targets that produces siRNAs in *pel1* and *ski2* mutants is not the same, even if there is some overlap. How could loss of PEL1 function stimulate miRNA-induced siRNA production? And in particular, what is the explanation for enhanced siRNA production in *pel1* mutants not only of siRNAs mapping to 5′-cleavage fragments, but also to 3′-cleavage fragments as shown by the miR472-*RSG2* example? Several observations indicate the importance of ribosome association for miRNA-triggered siRNA production from *TAS* precursors. Ribosome profiling experiments demonstrate ribosome association with *TAS3* precursors (89,90), and the *TAS2* precursor contains a short open reading frame important for tasiRNA biogenesis, and both the precursor and cleavage fragments associate with polyribosomes (91). Furthermore, detailed biochemical analyses reveal the importance of ribosome stalling in proximity to the 5′ miR390 site in *TAS3* precursors (90), and ribosome stalling and collision at rare codons in transposable element mRNAs correlates with their production of 21-nt siRNAs (58) (observed in mutants defective in DNA methylation, and, therefore, often referred to as epigenetically activated siRNAs, or easiRNAs (28)). It appears, therefore, that stalled ribosomes, in particular in combination with RISC, act as a trigger of secondary siRNA production, perhaps by extending the time of RISC association with target mRNA by AGO interaction. Indeed, AGO proteins, including *Arabidopsis* AGO1, associate with polyribosomes and co-purify with ribosomal proteins (92–94). Thus, the subunit dissociation activity on stalled ribosomes of Dom34/Pelota and Hbs1 (55,95) could directly limit secondary siRNA formation from miRNA-target mRNAs associated with RISC. This proposition is consistent with all of the properties of enhanced miRNA-induced siRNA production in *pel1* mutants that we observe here, in particular the distinct set of miRNA targets affected compared with *ski2* mutants, and enhanced production of siRNAs mapping to 3′-cleavage fragments in some instances. We note that this model requires plant PEL1 to be able to cause splitting of subunits from ribosomes stalled at internal sites in mRNAs. It is at present unclear to what extent internal ribosome stalls are resolved by Pelota:Hbs1 (46,48,55,95), particularly because Pelota binding to stalled ribosomes is favored by A-sites free of mRNA (55,96). Nonetheless, cryo-EM structures of Pelota bound to stalled ribosomes with mRNA sequence downstream of the P-site indicated mRNA displacement from the channel upon Pelota binding, thus strongly suggesting that an empty A-site is not an absolute requirement for Pelota binding (97). In addition, as noted by D’Orazio and Green (46), the observation that loss of the mouse Hbs1 homologue GTPBP2 leads to neurodegeneration in a strain carrying an inactivating mutation in a brain-specific Arg-tRNA provides an example of requirement of resolution of internal ribosome stalls *in vivo* by Pelota:Hbs1 (98). Thus, although a direct implication of stalled ribosome eviction by plant PEL1 in limitation of secondary siRNA production is not uniformly supported by biochemical analysis of Pelota/Dom34 activity on stalled ribosomes *in vitro*, this proposition is consistent with other analyses of molecular properties of Hbs1:Pelota and their function *in vivo*.

### Possible reasons for ARGONAUTE specialization in secondary siRNA formation

The most highly conserved example of miRNA-induced secondary siRNA production in plants is the formation of AUXIN RESPONSE FACTOR (ARF)-targeting *TAS3* siRNAs by miR390. These tasiRNAs are fundamental for leaf and flower development, as documented by consequences of their loss in mutants required specifically for siRNA amplification. Defects in these mutants include aberrant developmental timing (99), and aberrant leaf and flower morphogenesis in multiple species (100–105). *TAS3* siRNAs are initiated by a conserved AGO7-miR390 RISC (106), not by an AGO1-based RISC, and it has not been clearly established why a specialized AGO protein is required for this process of secondary siRNA formation. Our analysis of illicit secondary siRNAs produced by AGO1-miRNA targets may reveal part of the answer to this mystery: in most angiosperms, *TAS3* siRNAs derive from the 5′-cleavage fragment of the *TAS3* precursor (107), by dicing from its 3′-end following miR390-guided cleavage and conversion into dsRNA. The 5′-cleavage fragment must therefore maintain a well-defined 3′-end to produce functional ARF-targeting siRNAs in phase with the miR390-guided cleavage site. However, AGO1-miR166 induced secondary siRNAs mapping to *PHB*, and AGO1-miR319 induced secondary siRNAs mapping to *TCP2* were completely unphased, at least in part due to tailing and nibbling of the 3′-end of the 5′-cleavage fragment. Strong tailing and trimming activities are associated with AGO1, but less so with AGO7, as suggested by the different effect of mutation of the small RNA methyl transferase HEN1 on AGO1-bound miR173 (and other miRNAs) and AGO7-bound miR390. In *hen1* mutants, AGO1-bound miRNAs decrease dramatically in abundance and accumulate as a distribution of 17–27 nt species, while miR390 has reduced abundance, but largely maintains a 21-nt size (108). Thus, employment of a specialized AGO protein, AGO7, may help maintain the crucial phased register of *TAS3* siRNAs. We note, however, that this cannot be the only specialization of AGO7 relevant for *TAS3* siRNA biogenesis, as previous analyses also pointed to the requirement of AGO7-specific activities at the non-cleavable miR390 site in *TAS3* precursors (106).

## DATA AVAILABILITY

The sequencing data generated in this publication have been deposited in NCBI’s Gene Expression Omnibus (https://www.ncbi.nlm.nih.gov/geo/query/acc.cgi?acc=GSE173426).

R code is available in the GitHub repository https://github.com/MariaLouisaVigh/ExosomePelota.

Small RNA sequencing data are accessible through GEO Series accession number GSE173426.

## Supplementary Material

gkab1289_Supplemental_Files
