# Supplementary material for: Nuclear and cytoplasmic RNA exosomes and PELOTA1 prevent miRNA-induced secondary siRNA production in Arabidopsis
Source: Nucleic Acids Res. 2022 Jan 17;50(3):1396–415. doi: 10.1093/nar/gkab1289 (PMC8860578; doi:10.1093/nar/gkab1289)
Supplement: gkab1289_Supplemental_Files [file gkab1289_supplemental_files.zip › SUPPLEMENTARY MATERIAL.docx]

**SUPPLEMENTARY MATERIAL**

This article contains supplementary data:

Supplementary Figure 1: **Validation of small RNA sequencing data prior to DESeq analyses.**

Supplementary Figure 2: **Robustness of *ski2-5* samples across sequencing libraries.**

Supplementary Figure 3: **miRNA expression levels in mutants used in this study compared to Col-0 WT**.

Supplementary Figure 4: **miRNA-triggered siRNA accumulation in *ski2-5* and *pel1* mutants**

Supplementary Figure 5: ***ski2/pel1* double mutants are lethal**

Supplementary Figure 6: **siRNA accumulation in *sop1-5* and *rrp45a* mutants**.

Supplementary Figure 7: **siRNA production from miRNA targets in *ski2-5*, *hen2-5* and *rrp4-2***

Supplementary Table 1: **List of oligonucleotides used in the study**

Supplementary Table 2: **List of genes with significant different levels of sRNAs in *ski2-5, ski3-5* and *rrp45b* (Experiment A)**

Supplementary Table 3: **List of genes with significant different levels of sRNAs in *ski2-5, pel1-1, pel1-2, hen2-5, rrp4, rrp45a* (Experiment B)**

Supplementary Table 4: **List of genes with significant different levels of sRNAs in *ski2-5, hen2-5* and *sop1-5* (Experiment C)**

Supplementary Table 5: **List of known miRNA targets (experimentally verified)**

Supplementary Table 6: **List of binding energies in miRNA:target pairs (seed region)**
