# Supplementary material for: Nuclear and cytoplasmic RNA exosomes and PELOTA1 prevent miRNA-induced secondary siRNA production in Arabidopsis
Source: Nucleic Acids Res. 2022 Jan 17;50(3):1396–415. doi: 10.1093/nar/gkab1289 (PMC8860578; doi:10.1093/nar/gkab1289)
Supplement: gkab1289_Supplemental_Files [file gkab1289_supplemental_files.zip › Supplementary_Data.pdf]

Figure S1.

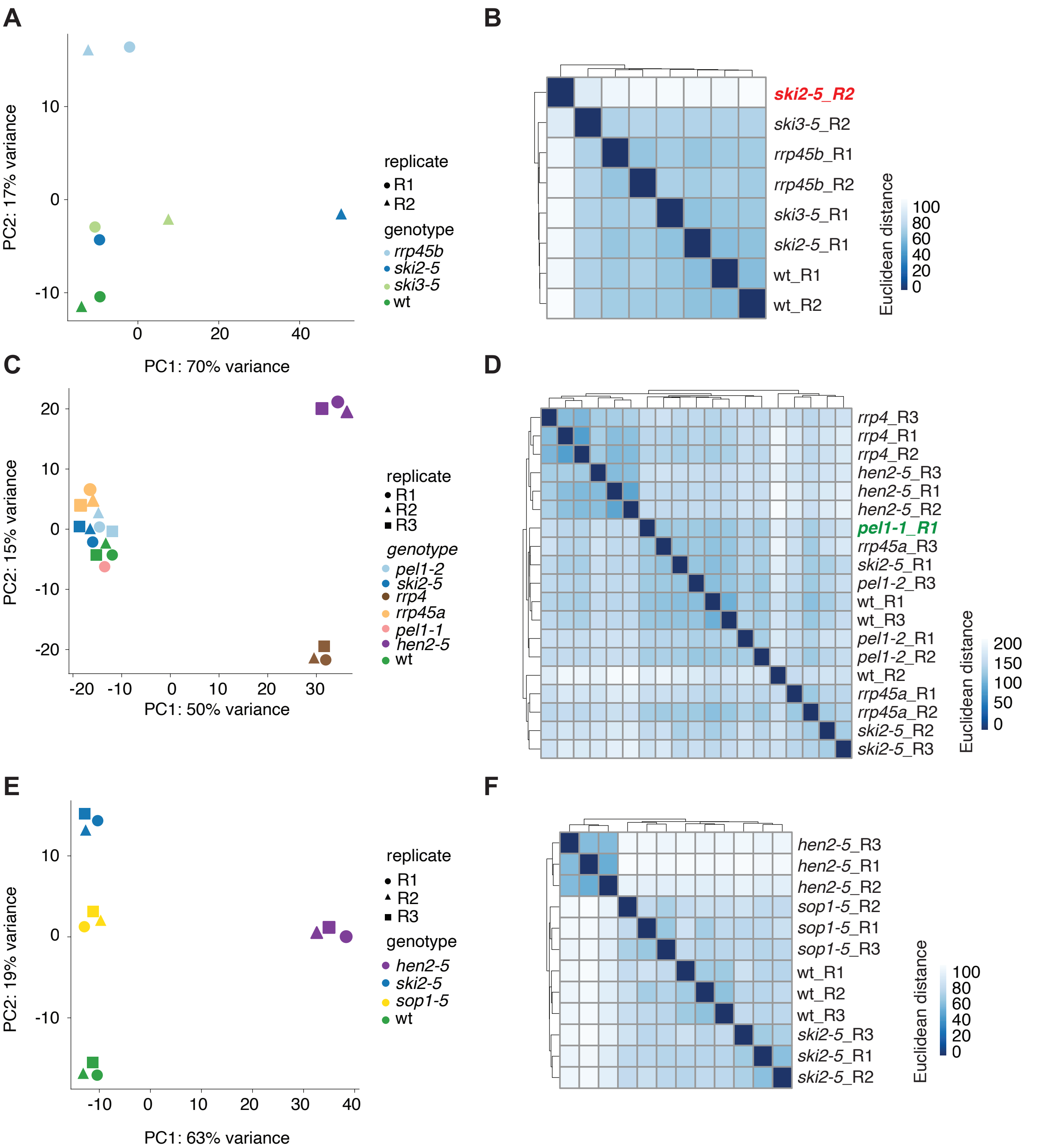

Supplemental Figure 1. Validation of small RNA sequencing data prior to DESeq analyses.
(A) and **(B)**, small RNA sequencing experiment A, used for analyses reported in Figure 2; **(C)** and **(D)**, small RNA sequencing experiment B, used for analyses reported in Figures 3-7; **(E)** and **(F),** small RNA sequencing experiment C, used for analyses reported Supplemental Figure 6.
(A) Principal component 1 and 2 of WT, *ski2-5, ski3-5* and *rrp45b*. The genotypes are distinguished by color and the two biological replicates are distinguished by shapes. **(B)** A distance matrix of WT, *ski2-5, ski3-5* and *rrp45b.* The second biological replicate of *ski2-5* is written in red to indicate that it is an outlier. Based on the principal component analysis and the distance matrix, this replicate of *ski2-5* was excluded from the DESeq analysis. The first replicate of *ski2-5* was still used for plotting of sRNA reads on miRNA target coordinates in figure 2C. **(C)** Principal component 1 and 2 of WT, *ski2-5, pel1-1, pel1-2, rrp4, rrp45a* and *hen2-5*. The genotypes are distinguished by color and the biological triplicates in the pool of libraries are distinguished by shapes. **(D)** A distance matrix of WT, *ski2-5, pel1-1, pel1-2, rrp4, rrp45a* and *hen2-5.* The only sample of *pel1-1* is written in green as it clusters together with the *pel1-2* samples. *pel1-1* was excluded from downstream DESeq analysis, but was used for plotting of sRNA reads on miRNA target coordinates in figure 2B.
(E) Principal component 1 and 2 of WT, *ski2-5, hen2-5* and *sop1-5.* The genotypes are distinguished by color and the biological triplicates in the pool of libraries are distinguished by shapes. **(F)** A distance matrix of WT, *ski2-5, hen2-5* and *sop1-5.*

Figure S2.

A

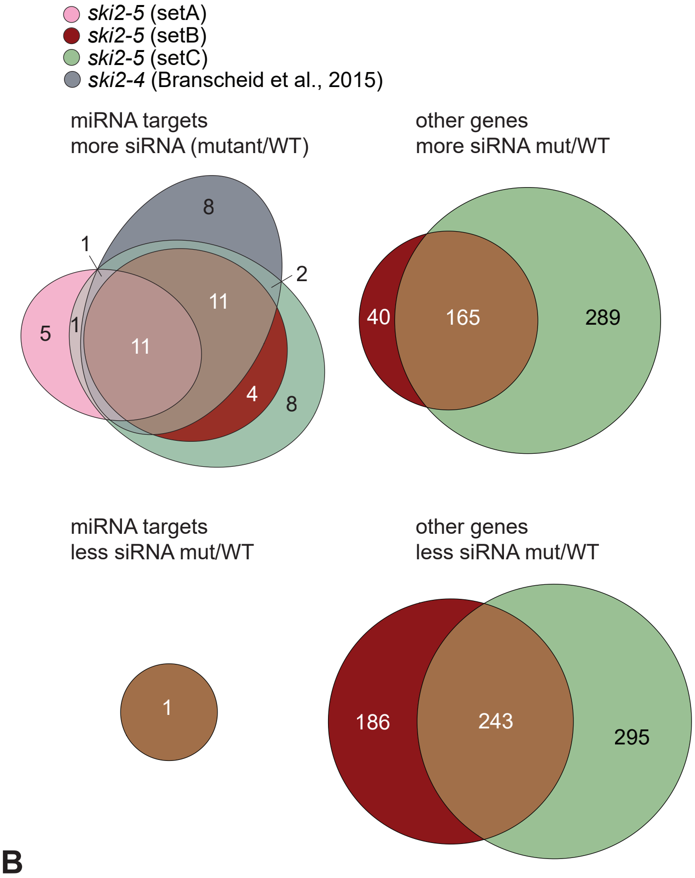

B

	ski2-5 (setA)	*ski2-5* (setB)	*ski2-5* (setC)	*ski2-4* (Branscheid et al., 2015)
ARF10	√	√	√	√
AGO1	√	√	√	√
PHB	√	√	√	√
TCP4	√	√	√	√
PHO2	√	√	√	√
SPL2	%	%	√	√
RSG2	%	%	√	%
TCP2	√	√	√	√

Supplemental Figure 2. Robustness of ski2-5 samples across sequencing libraries (A) Overlap in genes with significantly different levels of sRNAs in *ski2-5* compared to WT in sequencing experiments A, B and C. miRNA target genes in *ski2-5* (SetA) were filtered based on log_2FC values > 1 instead of p_{adj} due to its lack of biological replicates. miRNA target genes in *ski2-4* were retrieved from Table S2 *(Branscheid et al., 2015)* **(B)** Overview of miRNA targets used as examples in Figure 2, 3 and 5 and which of our datasets these have more siRNAs in *ski2*/WT.

Figure S3.

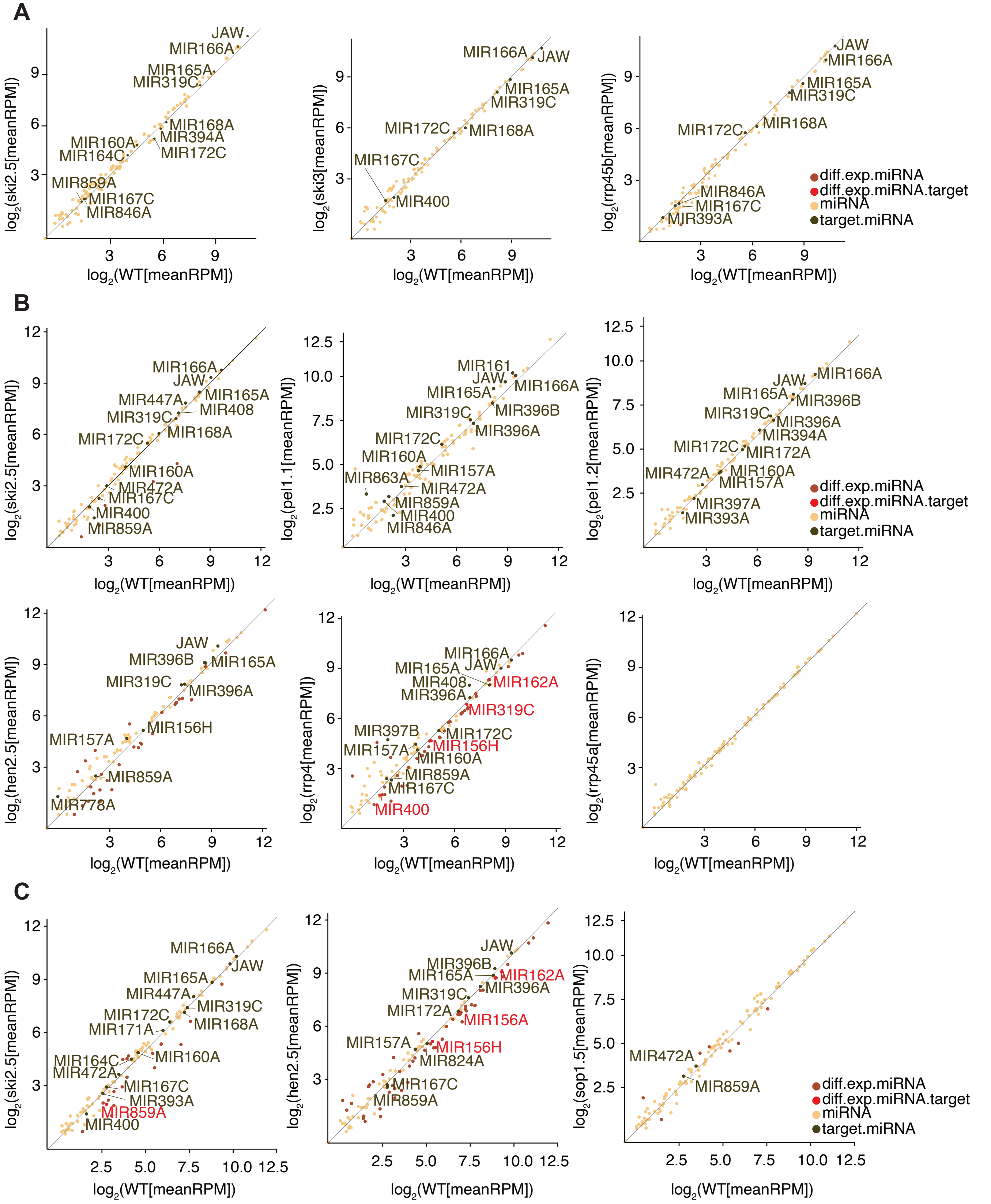

Supplemental Figure 3. miRNA expression levels in mutants used in this study compared to Col-0 WT. **(A,B,C)** Scatter plots showing mean RPM of all miRNAs in mutants compared to Col-0 wild type. Black, miRNA with mRNA targets that give rise to different siRNA levels compared to wild type; yellow, miRNA with mRNA targets with no apparent difference in siRNA levels compared to wild type; brown, differentially expressed miRNA; red, differentially expressed miRNA with mRNA targets that give rise to different siRNA levels compared to wild type.

(A) Experiment A, miRNAs in *ski2-5, ski3-5* and *rrp45b* compared to Col-0 WT. Targets with ectopic secondary siRNA production in *ski2-5* were filtered based on $\log_2 FC > 1$ instead of $p_{adj} < 0.05$ due to lack of replicates. **(B)** Experiment B, miRNAs in *ski2-5, pel1-1, pel1-2, hen2-5, rrp4* and *rrp45b* compared to Col-0 WT. Targets with ectopic secondary siRNA production in *pel1-1* were filtered based on $\log_2 FC > 0.9$ instead of $p_{adj} < 0.05$ due to lack of library replicates. **(C)** Experiment C, miRNAs in *ski2-5, hen2-5* and *sop1-5* compared to Col-0 WT.

Figure S4.

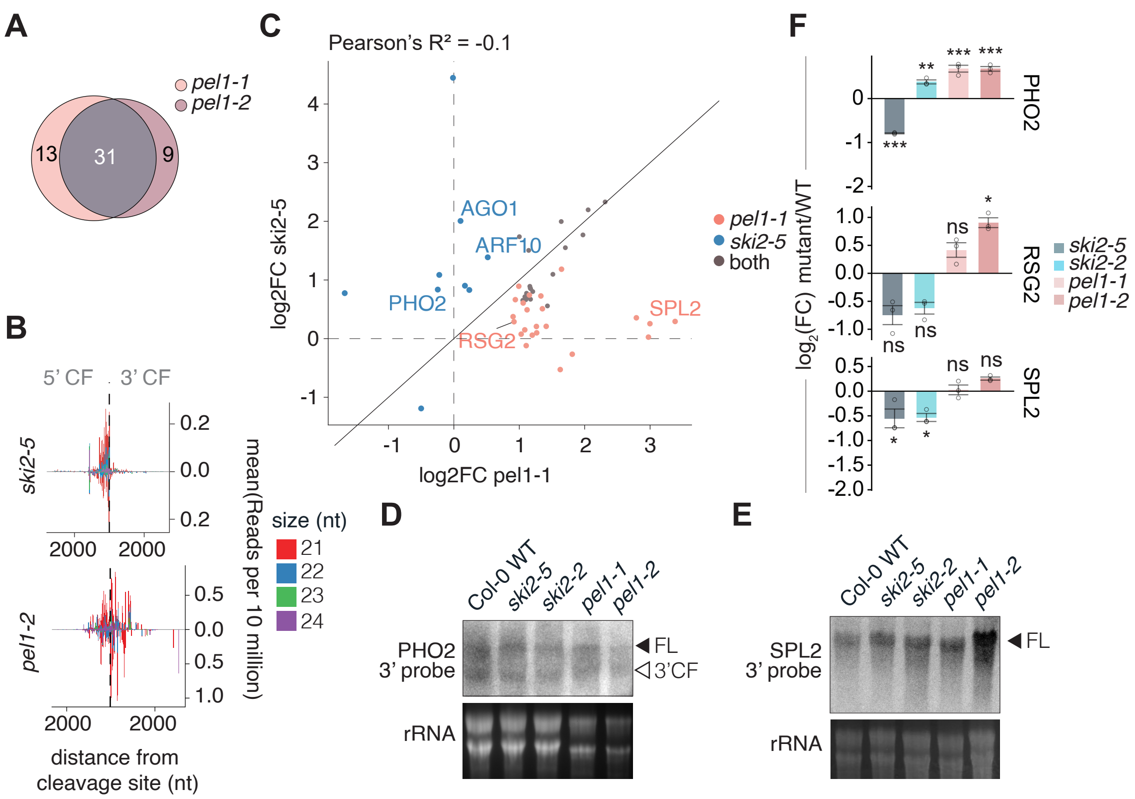

Supplemental Figure 4. miRNA-triggered siRNA accumulation in *ski2-5* and *pel1* mutants
(A) Overlap in miRNA-target genes with more siRNA-production (mutant/WT) between *pel1-1* and *pel1-2***.**
Due to lack of biological replicates in *pel1-1,* miRNA targets were considered to produce more siRNAs than
WT when $log_2FC > 0.9$. Targets in *pel1-2* have significantly higher levels of siRNAs in mutant compared to WT (filtered on $p_{adj} < 0.05$ in DESeq2 analysis). **(B)** Metaplot of siRNA read densities (RP10M) along miRNA target transcripts with significantly higher siRNA production in mutants than in wild type. Position 0 is defined by miRNA-guided cleavage sites. **(C)** Scatter plot of log_2 of the fold change of read densities (RPM(mutant)/RPM(WT)) of siRNAs (log_2FC) mapped to miRNA targets in *pel1-1* (x-axis) and *ski2-5* (y-axis). Only miRNA targets with higher siRNA read counts in either mutant compared to Col-0 are included. miRNA targets shown in Figure 3B are indicated. **(D)** and **(E)** Northern blots showing abundance of PHO2 and SPL2 mRNA in *ski2-5, ski2-2, pel1-1* and *pel1-2.* 20 µg of total RNA was loaded on two gels for Northern blotting. Ethidium bromide-stained ribosomal RNA is used as a loading control. One membrane was hybridized to a 3'-CF-specific PHO2 probe (D), resulting in detection of both full length (~4200 nt) and 3'-CF (~ 3300 nt). The other membrane was hybridized to a 3'-CF-specific SPL2 probe (E), resulting in detection of only full length SPL2 mRNA (~1700 nt). The more intense SPL2 signal in *pel1-2* is due to overloading of the lane as seen by EtBr-stained rRNA. **(F)** qPCR analysis was also performed to assess full-length mRNA abundance in *ski2-5, ski2-2, pel1-1* and *pel1-2.* 2 µg of the same total RNA used in (D) and (E) was used to make cDNA, and qPCR primers defining amplicons spanning miRNA-guided cleavage sites were used in all cases to detect only uncleaved mRNA. The average log_2FC (mutant/WT) of three technical replicates is plotted for PHO2, RSG2 and SPL2 and the error bars indicate s.e.m. Statistical differences in expression level between mutants and WT were tested with a Tukey test for each target. Indication of significance is ***$P <$ 0.001, **$P < 0.01$, *$P < 0.05$, ns = not significant.

Figure S5.

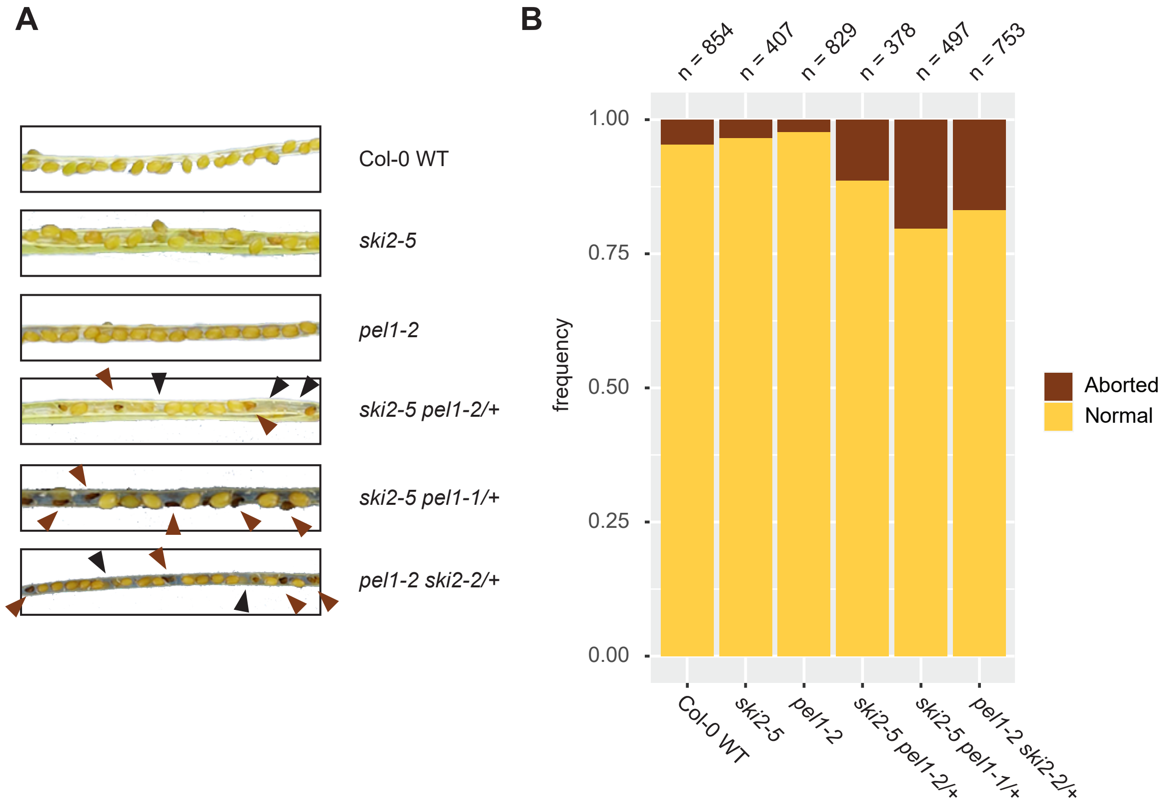

Supplemental Figure 5. *ski2/pel1* double mutants are lethal
(A) Representative pictures of siliques of Col-0 WT, *ski2-5, pel1-2, ski2-5 pel1-2/+*, *ski2-5 pel1-1/+* and *pel1-2 ski2-2/+*. The siliques of the latter three have approximately 25% of aborted seeds. The aborted seeds are either observed as empty spots in the silique (black arrows) or as dark-brown, deformed seeds small in size (brown arrows). The siliques were fixed with tape on a blue surface for photographing, this blue background was removed from the pictures in Photoshop. **(B)** Ratios of aborted/healthy seeds from the plants analyzed in (A). The number of seeds counted from each plant (n) is written above the plot.

Figure S6.

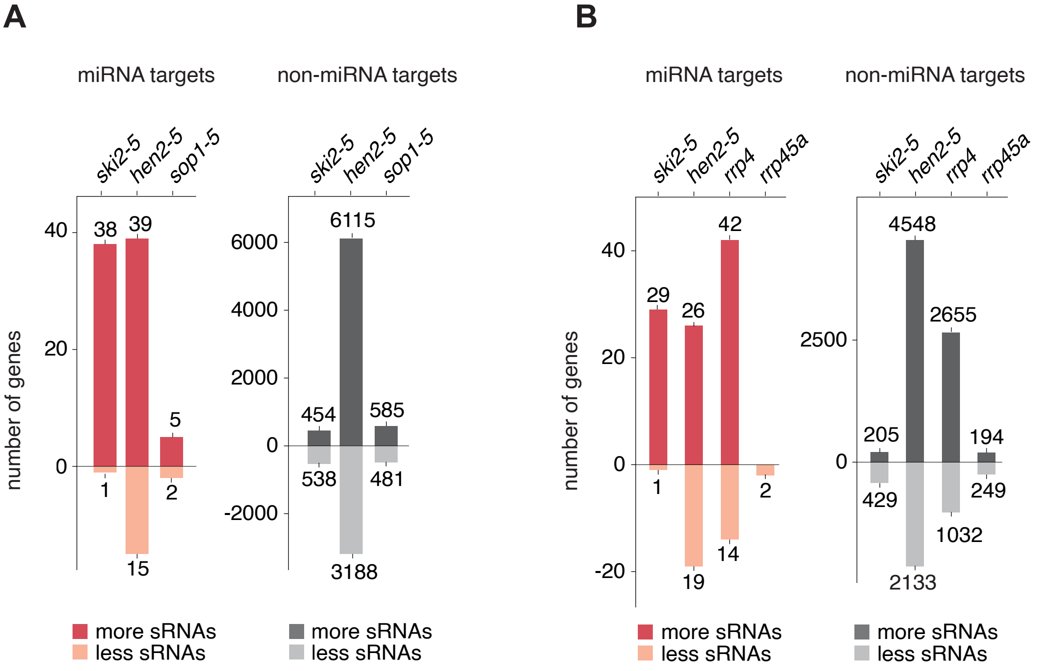

Supplemental Figure 6. siRNA accumulation in *rrp45a* and *sop1-5* mutants.
(A) Bar plots depicting number of known miRNA targets (red bars) or number of non-miRNA targets (grey bars), which produce either more or less secondary siRNAs in *ski2-5, hen2-5* or *sop1-5* compared to Col-0 WT (Wald test, $P < 0.05$). The enrichment of miRNA targets in genes producing more siRNAs is highly significant in *ski2-5* (Fisher-test: ***$P < 2.2 \times 10^{-16}$). In contrast, the proportion of miRNA targets found in genes with lower levels of siRNAs in the *ski2-5* compared to WT is not significant (Fisher-test: $P = 0.14$). The enrichment of miRNA targets in genes producing more siRNAs is not significant in *hen2-5,* nor in *sop1-5* (Fisher-test for *hen2-5*: $P = 0.20$, Fisher-test for *sop1-5*: $P = 0.81$). The proportion of miRNA targets found in genes with lower levels of siRNAs in the *hen2-5* and *sop1-5* mutants compared to WT is also not highly significant (Fisher-test for *hen2-5*: *$P = 0.043$, Fisher-test for *sop1-5*: $P = 0.60$). **(B)** Bar plots depicting number of known miRNA targets (red bars) or number of non-miRNA targets (grey bars), which produce either more or less secondary siRNAs in *ski2-5, hen2-5, rrp4-2* or *rrp45a* compared to Col-0 WT (Wald test, $P < 0.05$). The enrichment of miRNA targets in genes producing more siRNAs is significant in *ski2-5* and *rrp4* (Fisher-test for *ski2-5*: ***$P < 2.2 \times 10^{-16}$, Fisher-test for *rrp4-2*: ***$P = 1.7 \times 10^{-4}$). In contrast, the proportion of miRNA targets found in genes with lower levels of siRNAs in the *ski2-5* and *rrp4* mutants compared to WT is not significant (Fisher-test for *ski2-5*: $P = 1.0$, Fisher-test for *rrp4*: $P = 0.076$). The enrichment of miRNA targets in genes producing more siRNAs is not significant in *hen2-5* and *rrp45a* (Fisher-test for *hen2-5*: $P = 0.12$, Fisher-test for *rrp45a*: $P = 0.41$). This is the same for the proportion of miRNA targets found in genes with lower levels of siRNAs in the *hen2-5* and *rrp45a* mutants compared to WT (Fisher-test for *hen2-5*: $P = 0.62$, Fisher-test for *rrp45a*: $P = 1.0$).

Figure S7.

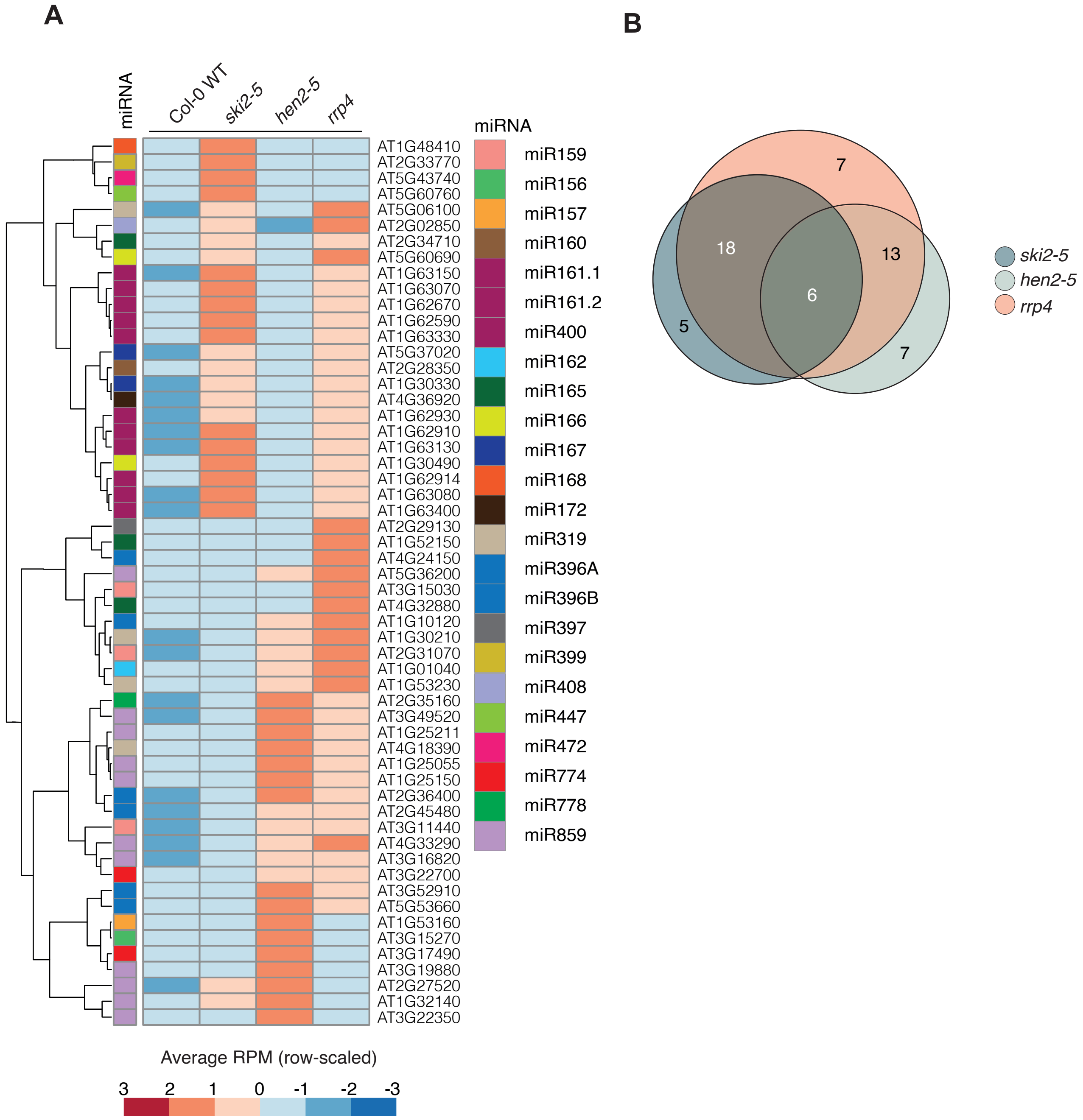

Supplemental Figure 7. siRNA production from miRNA targets in *ski2-5, hen2-5* and *rrp4-2*
(A) Heatmap of known miRNA targets with significantly more siRNAs produced in *ski2-5, hen2-5* and *rrp4-2* compared to WT ($p_{adj} < 0.05$). In the heatmap, the z-score of the mean RPM of siRNAs mapped to each miRNA target in WT, *ski2-5, hen2-5* and *rrp4-2* are used. The heatmap is clustered by targets. The identity of miRNA targets represented is indicated to the right, and miRNAs known to cleave the corresponding targets are indicated with a color to the left of each row. **(B)** Euler diagrams showing overlap in siRNA-producing miRNA targets between *ski2-5, hen2-5* and *rrp4-2* mutants

SUPPLEMENTARY TABLE S1

Oligoname	Sequence (5' to 3')	Purpose
ski2-2_Salk_LP	AGGTATCTCAATGTCCGGACC	Genotyping PCR
ski2-2_Salk_RP	ACACAACCGTGGCACTTATTC	Genotyping PCR
ski2-5_Salk_LP	GAACTGGTCTTTTTGTCGTGC	Genotyping PCR
ski2-5_Salk_RP	TAAATTTGCGGACATTTGAGG	Genotyping PCR
ski3-5_GK_LP	AGATGAGGCTTTTGAGAGTT	Genotyping PCR
ski3-5_GK_RP	ATTAGCGCATTCCATAACAGATTC	Genotyping PCR
ski8-1_Salk_LP	ACAGAGAGACCACGAGAGCAG	Genotyping PCR
ski8-1_Salk_RP	GAAGCAAATAAAAACTCCACTGC	Genotyping PCR
pel1-1_Sail_LP	GAGAAGCTGTGGAACGAATC	Genotyping PCR
pel1-1_Sail_RP	GGCATACCAAGCCCTTAG	Genotyping PCR
pel1-2_GK_LP	CGAGTCTGTATGTTCCTTCAC	Genotyping PCR
pel1-2_GK_RP	GCATGGAGAACCTCACC	Genotyping PCR
rrp45a_GK_LP	CGTGATCATACATCCACCCGAAG	Genotyping PCR
rrp45a_GK_RP	ATGCCGCTTCACCTTCTGTG	Genotyping PCR
rrp45b(cer7-3)_Sail_LP	CTGGCTGTTCTGGTTGGAGT	Genotyping PCR
rrp45b(cer7-3)_Sail_RP	CATTTCCAGAGCCGTTCATT	Genotyping PCR
hen2-4_Salk_LP	TATGGTATTCAGCAACCTCCG	Genotyping PCR
hen2-4_Salk_RP	GTTCCTCAAATGCTGCTCTTG	Genotyping PCR
hen2-5_GK_LP	GACTTGTGAAAGCGCTTTTTG	Genotyping PCR
hen2-5_GK_RP	TATGGTATTCAGCAACCTCCG	Genotyping PCR
sop1-5_Salk_LP	GGCGAGCAATGAGTTGAATCG	Genotyping PCR
sop1-5_Salk_RP	ACTTCGCCAACACCTTATCACC	Genotyping PCR
TDNA_GABI08474	ATAATAACGCTGCGGACATCTACATTTT	Genotyping PCR
Salk_TDNA_Lbb1.3	ATTTTGCCGATTTCGGAAC	Genotyping PCR
Sail_TDNA_LB3	TAGCATCTGAATTTCATAACCAATCTCGATACAC	Genotyping PCR
rrp4-2(SOP2)_Eco47I_F	CTATTCCCGTCAACCATGACG	Genotyping PCR
rrp4-2(SOP2)_Eco47I_R	CATCGACCTCGGAAGTTCCATGT	Genotyping PCR
rdr6-12 BfaI F	TGCAAGAGGAACGTGTGAGGTG	Genotyping PCR
rdr6-12 BfaI R	GCTTCAACCTCTTGTACGCATC	Genotyping PCR
AGO1_5'_F	AGAGAAGAACGATGCTCCA	probe
AGO1_5'_R	TTGTTGCTGTTGTGGTGGTT	probe
AGO1_3'_F	GGATTTGCACCATTATGAT	probe
AGO1_3'_R	TCAAGAACCTGCAGAGCTT	probe
ARF10_5'_F	TATCCTCTGTCGTGTCGT	probe
ARF10_5'_R	GAACGGAGGAAGACGATTGA	probe
ARF10_3'_F	GTGTTCTTTAACTATGGGG	probe
ARF10_3'_R	TAACACCATTTGCATCCCG	probe
CSD2_5'_F	CCAAACGTCAAACATAGCAGCA	probe
CSD2_3'_R	CCGCGGAAACAACTGTCAAC	probe
CSD2_5'_F	GGGTGACCTGGGAAACATAA	probe
CSD2_3'_R	TCAAGCCAATCACACCACAT	probe
PHO2_3'_F	GAGCACCCTGATTTTGGATTCTG	probe
PHO2_3'_R	TCCTGATGGTTTGAAGATGCTTCA	probe
SPL2_3'_F	TCAAGCAACCTCCAACCC	probe

SPL2_3'_R	GAGGATTTGGTGTCTTGC	probe
PHO2_FL_F	GCAGCAGAAGTGAAGTTTCT	qPCR
PHO2_FL_R	GAGATTTGCCCAACGAATG	qPCR
RSG2_FLand3CF_F	GATGTAGTGATATGGGTTGT	qPCR
RSG2_FLand3CF_R	CTACTTCGCTCCAGAGATCA	qPCR
SPL2_FL_F	CACACATGGGTGCTTCTCAA	qPCR
SPL2_FL_R	AAGGGTAAAACGCCTTGGTT	qPCR

SUPPLEMENTARY TABLE S1. Oligonucleotides used in the study.
